# Nuclear receptors in health and disease: signaling pathways, biological functions and pharmaceutical interventions

**DOI:** 10.1038/s41392-025-02270-3

**Published:** 2025-07-28

**Authors:** Ping Jin, Xirui Duan, Zhao Huang, Yuan Dong, Jianmei Zhu, Huiming Guo, Hui Tian, Cheng-Gang Zou, Ke Xie

**Affiliations:** 1 State Key Laboratory for Conservation and Utilization of Bio-Resources in Yunnan and Key Laboratory of Industrial Microbial Fermentation Engineering of Yunnan Province, School of Life Sciences, Yunnan University, Kunming, China; 2 Department of Oncology, Ruijin Hospital, Shanghai Jiao Tong University, School of Medicine; Shanghai Key Laboratory of Gastric Neoplasms, Shanghai, China; 3 West China Institute of Preventive and Medical Integration for Major Diseases, West China School of Public Health and West China Fourth Hospital, Sichuan University, Chengdu, China; 4 Department of Breast Surgery, the First Affiliated Hospital of Kunming Medical University, Kunming, China; 5 Department of oncology, Sichuan Provincial People’s Hospital, University of Electronic Science and Technology of China, Chengdu, P.R. China; 6 Department of Gynecology, the First Affiliated Hospital of Kunming Medical University, Kunming, China; 7 Department of Radiation Oncology, the First Affiliated Hospital of Kunming Medical University, Kunming, China

**Keywords:** Metabolic disorders, Cardiovascular diseases, Cell biology, Cancer

## Abstract

Nuclear receptors (NRs) are a large family of ligand-dependent transcription factors that regulate the expression of a wide range of target genes in response to endogenous and exogenous ligands, including steroid hormones, thyroid hormone, vitamin D, retinoic acid, fatty acids, and oxidative steroids. Upon ligand binding, nuclear receptors form dimer complexes with transcriptional cofactors, which interact with specific DNA sequences in the promoter or enhancer regions of target genes to modulate gene expression. This process plays a crucial role in many physiological processes such as reproduction, development, immune responses, metabolism, and homeostasis. Dysregulation of nuclear receptor signaling is implicated in the pathogenesis of numerous diseases, including cancers, metabolic disorders, cardiovascular diseases, and autoimmune conditions. Therefore, understanding the molecular mechanisms underlying nuclear receptor functions is essential for the development of novel therapeutic strategies. This review summarizes the current understanding of nuclear receptors in both physiological and pathological contexts, providing insights into the signaling pathways they regulate. Additionally, we discuss recent advances in drug development targeting nuclear receptors, with a focus on preclinical and clinical studies aimed at improving therapeutic efficacy. By exploring these therapeutic avenues, this article highlights the potential of nuclear receptors as promising targets for future treatments of a variety of human diseases, paving the way for more personalized and effective therapies in clinical medicine.

## Introduction

A cell’s ability to detect various extracellular signals depends on the chemical nature of the signaling ligands and the permeability of the plasma membrane to polar compounds, such as polypeptides.^1,2^ This necessitates the production of two main types of signaling ligands and receptors. The first type, represented by growth factors, consists of polypeptides that generally cannot penetrate the cell membrane^3–5^; therefore, their receptors must present sophisticated extracellular binding domains. The second type of nuclear receptor ligand includes small, relatively hydrophobic molecules such as steroid hormones, vitamins, fatty acid derivatives, dexamethasone and enzalutamide.^6^ The hydrophobicity of these ligands allows them to pass through the cell membrane and reach the nucleus to bind to and activate nuclear DNA-binding proteins that then act as transcription factors.^7^

Nuclear receptors (NRs) are a class of ligand-activated transcription factors that sense certain molecules, such as steroids, thyroid hormones and vitamins, to directly bind DNA and modulate gene expression, bypassing the need for cytoplasmic signal cascades that mediate surface receptor and nuclear transcription mechanisms.^8–10^ They are essential for numerous physiological processes, including development, metabolism, reproduction, and homeostasis. To date, 48 NRs have been identified in the human genome, and 49 have been identified in mice. The typical structure of a nuclear receptor consists of a hinge region, a conserved ligand-binding domain and a DNA-binding domain, which are surrounded by various N- and C-terminal domains.^11^ While some nuclear receptors are physically connected to chromatin, others remain in the cytoplasm until ligand interactions allow nuclear entry. These receptors bind to a DNA recognition sequence in or close to the promoters of the genes they control once they are attached to chromatin, either as homodimers or heterodimers.^12,13^ These recognition sequences are commonly called hormone response elements (HREs) and are composed of two hexanucleotide sequences separated by a variable number of spacer sequences.^14^ In addition, NRs can be indirectly recruited to the genome by tethering mechanisms by other DNA-bound transcription factors.^15^ Several posttranslational modifications, such as phosphorylation, ubiquitination and SUMOylation, have been reported to modulate the activities of NRs.^16–19^

The regulation of gene expression by nuclear receptors influences the proliferation,^20^ differentiation^21^ and death^22,23^ of many different cell types, thereby playing important roles in the reproduction, development and physiology of metazoans.^24–27^ The characterization of the NR superfamily marked a significant turning point in the field of endocrine physiology by establishing common molecular pathways governing the development and physiology of various animal species. The dysregulation of these genes can lead to cardiovascular disease,^28^ cancer^29^ and diabetes.^30^ Research on NRs began in the mid-1980s when molecular cloning of several hormone receptors revealed their common structural framework.^31^ As a consequence of their central role in governing cell fate, NRs constitute targets for 15–20% of all pharmacologic drugs.^32,33^

Drugs that target specific NRs are frequently employed to treat a wide range of illnesses. Tamoxifen and raloxifene, for example, target the estrogen receptor (ER) and are used to treat osteoporosis and breast cancer.^34,35^ Enzalutamide is used to treat prostate cancer by targeting the androgen receptor (AR),^36,37^ whereas thiazolidinediones are used to treat type 2 diabetes by targeting peroxisome proliferator-activated receptor-gamma (PPARγ).^38,39^ Compounds with stronger binding affinities and better specificity are currently under development as new NR-targeted drugs because the available drugs often lack specificity and exhibit significant side effects, including severe heart failure.^40,41^ Thus, understanding the mechanism of NR gene selection and the development of specific inhibitors targeting NRs has become a pressing scientific issue in the field of biomedicine.

In this review, we systematically introduce NRs and their members, structure, functions and regulatory signals. We summarize recent findings regarding the function of NRs in regulating physiology and pathology and the relevance of these receptors to clinical drug development. Emphasis is given to research on the targeting of these receptors for disease management, the preclinical and clinical testing and development of novel drugs and the techniques and strategies that facilitate targeting and therapeutic efficacy.

## Overview of nuclear receptors

**Fig. 1:**
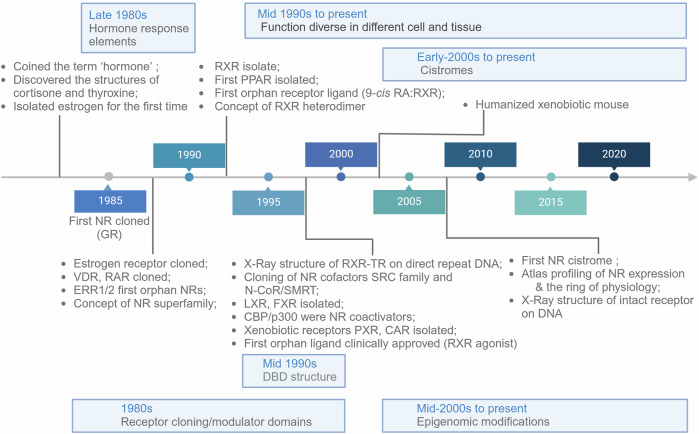
Historical timeline of nuclear receptor expression. Schematic timeline illustrating key milestones in the nuclear receptor research field. The timeline begins with the cloning of the first steroid hormone receptor cDNA and highlights significant advancements, culminating in recent discoveries enabled by “omics” technologies. Major breakthroughs and pivotal discoveries are annotated along the timeline

The discovery of hormones dates back to the early 1900s^42–45^ (Fig. 1). In 1905, Ernest Starling coined the term ‘hormone’.^44,45^ In 1926, Tadeus Reichstein and Edward Calvin Kendall elucidated the structures of cortisone and thyroxine,^46^ while Adolf Butenandt and Edward Adelbert Doisy first isolated estrogen in 1929.^47,48^ became clear only after Elwood Jensen conducted a series of experiments in the late 1950s to determine how estrogen regulates reproductive organ maturation.^49^ The rise of molecular biology in the 1980s led to groundbreaking advances, including the cloning of the human glucocorticoid receptor (GR) and the identification of the first estrogen receptor, ERα, from the ESR1 gene.^50–52^ In 1985, Ronald Evans successfully cloned the human glucocorticoid receptor (GR),^50,51^ while Pierre Chambon’s lab identified the first estrogen receptor, ERα, from the *ESR1*gene.^52^ They found that these receptors shared similarities with v-erbA, a viral oncogene recognized as a thyroid hormone receptor (TR) transcribed from the *THRA* gene. This discovery resulted in the placement of steroid and thyroid hormone receptors into a single grouping. A sequence comparison revealed a conserved evolutionary framework, highlighting structural and functional features that predicted the development of the nuclear receptor superfamily. Each receptor contains DNA-binding, ligand-binding, and transactivation domains. Soon after, receptors for other binding factors, such as retinoic acid and vitamin D, were cloned, confirming the existence of a receptor superfamily unified by structure. The formal establishment of the NR superfamily represented a major milestone, bolstering the hypothesis that the development and physiology of all animal species might be regulated by related molecular processes.

The therapeutic potential of NRs was recognized in the 1970s when it was shown that the estrogen blocker tamoxifen could inhibit ER-dependent breast cancer cells.^53,54^ In prostate cancer, multiple nuclear receptors have been shown to inhibit tumor growth, proliferation, and metastasis, leading to significant interest in targeting these receptors as therapeutic strategies.^55–57^ Dysfunction of NRs has been associated with specific diseases, such as infertility, obesity, and diabetes.^58^ As key drug targets, NRs are crucial in studying the pathological mechanisms of metabolic diseases and related drug development. Today, they represent a huge family of pharmaceutically targetable proteins. This review discusses the current knowledge on NR-based therapeutic intervention in different types of disease.

### Family members

The known sequence of the human genome revealed that forty-eight human NRs detect ligands, such as hormones, and translocate them into the nucleus to function as transcription factors regulating gene expression.^59^ These receptors are classified into three main types on the basis of their ligand types or into eight subgroups on the basis of sequence homology.^11,60^ Type I receptors are steroid receptors, including the ER, AR, progesterone receptor (PR), mineralocorticoid receptor (MR) and GR.^61,62^ Their ligands are steroid hormones such as sex hormones, glucocorticoids and mineralocorticoids.^63^ Type II receptors are nonsteroid receptors, such as thyroid hormones (TRα and TRβ), retinoic acid receptors (RARα, β), vitamin D receptors (VDRs) and peroxisome proliferator-activated receptors (PPARα, β and γ).^64^ Type III receptors include orphan receptors whose endogenous ligands are unknown or that do not bind to any ligands, such as the testicular receptor and germ cell nuclear factor.^65^ Alternatively, NRs can be divided into eight subgroups that are similar to classical receptors but differ enough individually, named thyroid hormone receptor-like family, retinoid X receptor-like family, estrogen receptor like family, nerve growth factor IB-like family, steroidogenic factor-like family, germ cell nuclear factor-like family, and those with two DNA-binding domains, as well as recently identified typically structured NR (CgNR8A1) in metazoans such as mollusks, annelids, cnidarians, echinoderms and hemichordates.^66,67^

### Structure

**Fig. 2:**
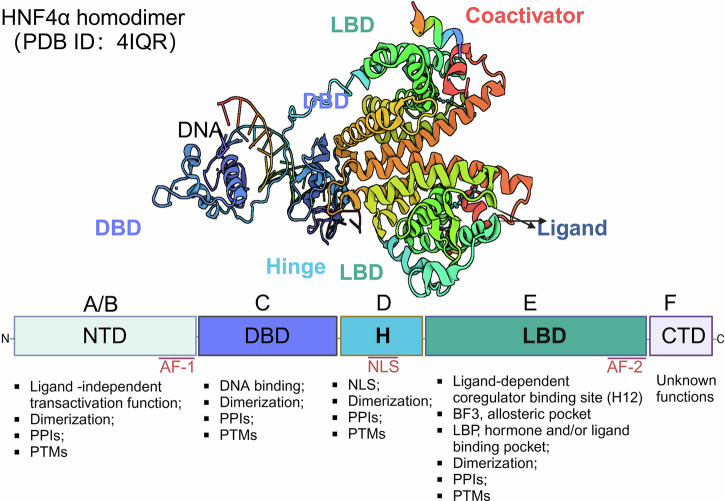
Diagram of the structural model of nuclear receptors. Nuclear receptors share four functional domains: the N-terminal transcription activation domain (NTD), the DNA binding domain (DBD), the ligand binding domain (LBD), and a hinge domain (H) that connects the DBD and LBD. Some NRs also contain a highly variable C-terminal transcription activation domain (CTD)

Except for the dose-sensitive sex reversal-adrenal hypoplasia congenital critical region on the X chromosome, gene 1 (DAX1) and the small heterodimer partner (SHP), all NRs are typically single-chain polypeptides that share four or five functional domains^,^^68^ including the N-terminal transcription activation domain (NTD), the DNA-binding domain (DBD), the ligand-binding domain (LBD), the C-terminal transcription activation domain (CTD) and a hinge domain (H) that connects the DBD and LBD (Fig. 2).

The NTD, which is located at the N-terminus and comprises 25–603 amino acids (AAs), contains the first of two transactivation regions (AF-1) and possesses transcriptional activator functions.^69,70^ The A/B domain is intrinsically disordered, which makes obtaining full-length 3D representations of the receptor difficult.^71,72^ The second identified subunit is the DBD, with 66--68 AAs, including two zinc fingers, which are typically obscured because of lower DNA affinity when the hormone is absent.^73–75^ Once a hormone is bound, the zinc fingers dock the hormone–receptor complex to hexanucleotide response elements located within NR-regulated promoters that determine the receptor’s regulatory specificity. The DBD also serves as an intermolecular interaction site for receptor dimerization.^76^ The LBD is located at the receptor’s C-terminus and contains 220--250 AAs.^77^ The LBD binds to the cognate hormone or ligand through an interior binding pocket.^78,79^ It also has an AF-2 site for recruiting various coactivating proteins, such as heat shock proteins (HSPs), to facilitate interactions with chromatin-remodeling proteins and the transactivation domain.^80–82^ The LBD plays a crucial role in mediating self-assembly reactions, such as dimerization or tetramerization, which are essential for high-affinity binding to DNA response elements.^83^ The DBD is connected to the LBD via a flexible domain, the ‘hinge’, which is related primarily to the nuclear localization signal of NRs and may influence the intracellular trafficking and subcellular distribution of the hormone‒receptor complex.^84,85^ The hinge region also contains a short NLS, and its phosphorylation is coupled to elevated transactivation.^86,87^

However, not all NRs have all four typical domains. Members of the NR0 subfamily, for example, frequently have neither a DBD nor an LBD in their structures.^88^ Among these, NRs with only the LBD and no DBD, such as Dax-1 and SHP, are nonetheless capable of binding to other transcription factors (TFs) and suppressing the expression of genes downstream. Finally, some NRs also possess a short, highly variable C-terminal domain (CTD or F domain) that often has unknown functions.^89,90^ However, current research has revealed a critical function of the F domain in the ERα reaction to select ER modulators (SERMs), particularly in SERM-mediated LBD dimerization and AF-1 activity.^91^

### Origins and evolution

NRs were thought to have evolved approximately 600 million years ago in the common ancestor of bilaterian animals, enabling them to sense and respond to internal and external signals.^92^ This evolution likely involved genetic amplification and mutation, contributing to the functional diversity of the NR superfamily.^93^ Most NR ligands are small, hydrophobic molecules that pass through cell membranes, although some, such as thyroid hormones, are actively transported into cells.^94^ While NRs were initially termed ligand-binding proteins, it remains debated whether their common ancestor bound ligands or functioned as constitutively active receptors. Over 50% of NRs are orphan receptors lacking known ligands, although some have been shown to bind metabolites, suggesting a role in sensing metabolic changes.^95,96^ The evolution of the LBD enabled interactions with small molecules and coregulators, increasing NR versatility.^97^ Gene duplication during early metazoan evolution expanded and specialized the NR superfamily into subfamilies such as steroid hormone receptors, thyroid hormone receptors, and orphan receptors. The coevolution of NRs and their ligands increases their physiological complexity. For example, vertebrate NRs, such as glucocorticoid and estrogen receptors, have evolved high specificity for steroid hormones, which are crucial for the stress response and reproduction.^98^

In 1992, a comparison of the DNA-binding domains of known NRs led to the construction of a phylogenetic tree, suggesting a common ancestor.^99^ By 1997, an alternative hypothesis emerged, suggesting that the original NR was an orphan receptor that gradually gained ligand-binding ability.^100^ The earliest NRs are believed to have evolved as orphan receptors that lack defined ligands and are involved in regulating fundamental processes such as metabolism and cellular signaling.^101^ Over time, orphan receptors adapt to regulate broader metabolic pathways, including lipid homeostasis and detoxification. A recent discovery of the NR7 subfamily has filled a critical gap in the understanding of the evolution of NR dimerization capabilities. Phylogenetically situated between class I NRs (monomers or homodimers) and class II NRs (heterodimers), the NR7 subfamily acts as a “missing link” in the transition from homodimerizing to heterodimerizing nuclear receptors.^102^

### Mechanisms of action

Nuclear receptors can modulate gene expression through direct binding to target genes as well as through interactions with other NRs.^103,104^ The interplay between NR actions can be described at progressively more detailed levels, with various regulatory modes involved in upstream events that influence downstream gene transcription and crosstalk events at the DNA level that directly influence gene transcription.^105^ The existence or lack of physical connections between the relevant NRs could further alter the crosstalk mechanisms at the DNA level.

#### Crosstalk between nuclear signalings

**Fig. 3:**
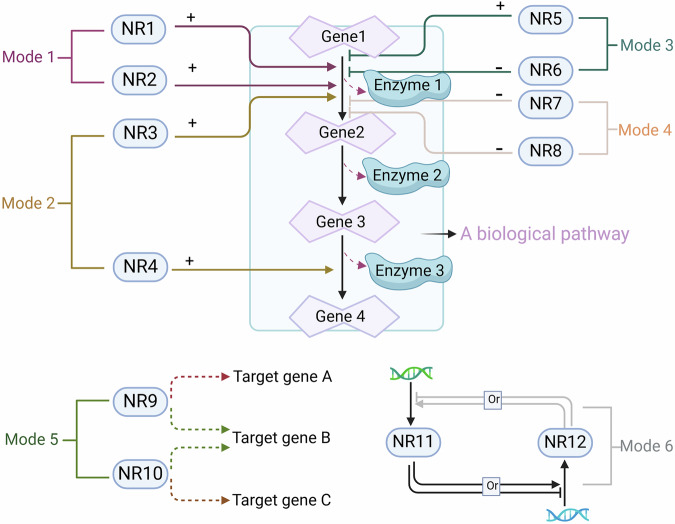
Crosstalk modes of NR signaling. Various regulatory modes involved in NR signaling influence downstream gene transcription. For example, different types of nuclear receptor signaling can simultaneously regulate the expression of the same gene (Modes 1, 3 and 4) or regulate different genes belonging to the same pathway (Mode 2). Different nuclear receptors may share the same target genes (Mode 5). In addition, crosstalk between two NRs can influence each other’s gene or protein expression (Mode 6)

Nuclear receptors can share overlapping arrays of target genes or regulate distinct genes that are part of the same downstream activity or pathway (Fig. 3). There is a wealth of experimental evidence to support endpoint modifications in gene expression that occur after NR crosstalk. The relationship between ERα and the PR was among the first observed examples of crosstalk between nuclear receptors.^106^ Estradiol-stimulated ERα activity was found to be suppressed by liganded PR isoforms, and the ligand, promoter and cell type were demonstrated to influence this suppressive crosstalk.^106,107^ Interactions between GRα and PPARα are critical in regulating shared subsets of target genes and different stages of signal transduction cascades involved in inflammatory and metabolic pathways.^108,109^ For example, both GRα and PPARγ have been shown to repress genes associated with TLR4- and TLR9-induced innate immune responses in primary peritoneal macrophages.^110,111^ However, there are significant differences in how these NRs achieve gene repression, which involves distinct transcriptional corepressor complexes. These variations in regulatory mechanisms highlight the complexity and specificity of gene regulation mediated by GRα and PPARγ in immune and metabolic contexts.

More complex types of crosstalk, such as reciprocal or unidirectional regulation of the expression of NR genes or proteins and ligand availability, can sometimes occur.^112,113^ For example, glucocorticoid-activated GRα was reported to upregulate PPARα mRNA expression.^109,114^ Conversely, fenofibrate-activated PPARα was shown to downregulate GRα expression at the posttranscriptional level in a time- and dose-dependent manner.^115,116^ In rodent adipocytes, antidiabetic PPARγ ligands increase the expression of PEPCK-C mRNA, which encodes the key glyceroneogenic enzyme EC4.1.1.32.^117^ In contrast, dexamethasone-activated GRα counteracted this induction at the transcriptional level. These results show that the processes involved in NR crosstalk are not unique; different pairs of nuclear receptors can interact through distinct crosstalk mechanisms according to the stimulus, expression level, cell type, pathway and genes involved. It is probable that many other modes of crosstalk will be uncovered as our knowledge expands to include other intracellular organelles and the shuttling of NRs.

#### Crosstalk between nuclear receptors at the DNA level

**Fig. 4:**
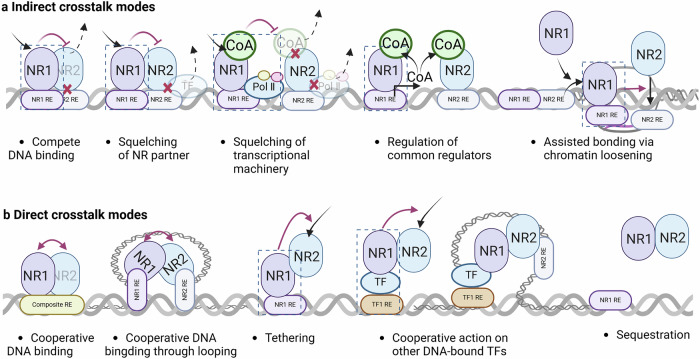
Crosstalk modes at the DNA level. At the DNA level, nuclear receptors engage in crosstalk by indirectly or directly interacting with DNA. **Part a** outlines indirect regulation modes, where no physical interaction occurs, whereas **Part b** describes regulatory mechanisms involving direct physical interactions among nuclear receptors (NRs). Indirect regulation modes include: NR signaling pathways may compete for overlapping DNA-binding sites; NR heterodimer partners may cause redistribution or squelching; Components of the transcriptional machinery, such as RNA polymerase II (Pol II) and transcriptional coregulators, may cause redistribution or squelching; The alteration of shared coregulators can influence the activity of other NRs. One NR family member can act as a pioneering factor to facilitate chromatin loosening and enable the subsequent binding of other NRs. Direct physical interactions includes: Paired receptors may cooperate for direct DNA binding or through DNA looping; Direct crosstalk can occur with just one partner contacting the DNA; Two nuclear receptors engage in crosstalk through protein-protein interactions with other transcription factors (TFs); The sequestration of one or both NRs (originating from other modes, (f–i) away from the DNA due to heterodimer interactions

At the DNA level, NR crosstalk can take two primary forms^13,118^ (Fig. 4). In one scenario, NRs physically interact to regulate specific target genes (direct crosstalk). In another context, NRs do not actually connect but still engage in unidirectional or bidirectional interactions (indirect crosstalk). When two nuclear receptors interact directly, they can bind to DNA sequences that are either closely positioned or farther apart. Alternatively, they can bind DNA sequences through the action of other transcription factors. A well-known example of this is the peroxisome proliferator-activated receptor PPARs, which are of three types: PPARα, PPARβ/δ and PPARγ. Most of these proteins are heterodimers connected to retinoid X receptors (RXRs) that regulate gene transcription,^119,120^ but they can also be fine-tuned by pairing with a different ligand. Multiple coactivators can interact with heterodimer complexes to supplement and stabilize their activity in regulating lipid metabolism, adipogenesis and inflammation.^121,122^ Further instances of direct NR connections have been reported for steroid receptors. GRα and AR have many target genes in common due to the recognition of partial palindromic repeats of the sequence 5′-TGTTCT-3′.^123^ GRα–AR heterodimerization has been proposed as the mechanism underlying the mutual inhibition of the transcriptional activities of these two receptors, allowing for differential regulation of specific genes.^124,125^ Furthermore, AR and GR share significant similarities in the dimerization structure of their LBDs, which sheds new light on the previously reported structure of the GR LBD.^126^ The mechanisms of direct crosstalk may allow for dual NR ligand control, but the exact conditions for this process have not been determined. Context-specific influences, such as tissue or cell type and differentiation status, are likely to lead to different interaction patterns, resulting in varied biological responses.^127,128^

In the absence of an actual connection, NR signaling pathways can indirectly influence crosstalk through several mechanisms.^129–131^ They may compete for overlapping DNA-binding sites by redistributing shared proteins or parts of the transcriptional machinery, such as RNA polymerase II or transcriptional coregulators; by regulating the expression of shared coregulators; and by acting as pioneering factors that loosen chromatin to facilitate the binding of additional NRs. For example, peroxisome proliferator-activated receptor alpha (PPARα) binds to estrogen-related receptor (ERR) target genes in heart tissue through ERR response element motifs via a histone deacetylase SIRT1-dependent mechanism.^132^ RXR, the PPAR partner, can trigger PPAR-independent activities on PPAR response element (RE)-controlled genes via RXR homodimerization, adding another layer of control to an intricate metabolic pathway.^133^ Members of the PPAR and ERR subfamilies can be further connected through shared coregulators, including PGC1α and PGC1β, as well as through common regulators such as low-energy-sensing AMP kinase.^134,135^ For example, during adipocyte differentiation and osteoclastogenesis, PGC1β, a coregulator that is upregulated by ERRα, can be recruited by PPARγ and other transcriptional regulators to enhance their adipogenic properties.^136^ Reduced PGC1β expression levels due to downregulation of ERRα resulted in decreased transcriptional activity of PPARγ. These reports provide evidence for the existence of complex combinations of indirect regulatory modes.

#### Genomic and nongenomic mechanisms

**Fig. 5:**
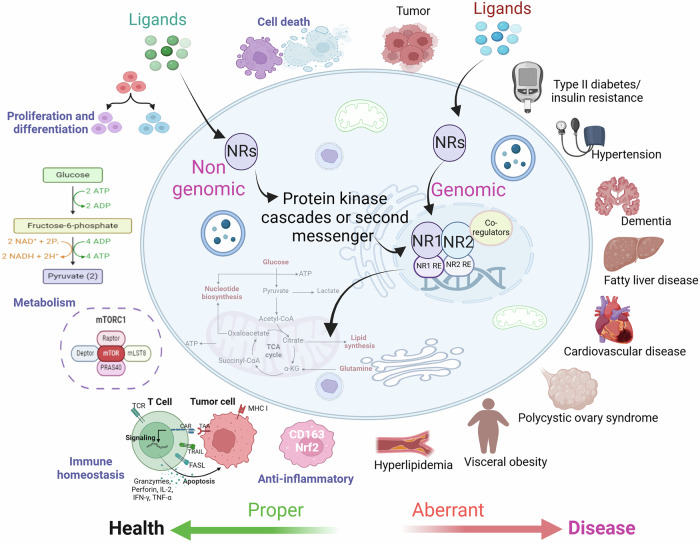
Nuclear receptors precisely regulate cell fate and metabolic processes. Nuclear receptors play crucial roles in regulating cell fate by influencing various cellular processes, including proliferation, differentiation, senescence and apoptosis, by acting as transcription factors. Additionally, members of the nuclear receptor family control the synthesis rates of different metabolic enzymes across various tissues, affecting processes such as glucose, lipid and bile acid metabolism, as well as adipocyte differentiation and plasma lipoprotein modulation. By regulating the balance between the storage, utilization and production of these macromolecules, nuclear receptors ensure metabolic flexibility, enabling the organism to adapt to fluctuations in energy availability and demand

The function of nuclear receptors can be achieved via genomic or nongenomic pathways (Fig. 5). Genomic mechanisms involve the direct regulation of gene expression through binding to DNA. This mechanism usually plays a critical role in long-term physiological processes, such as development, metabolism, and homeostasis. Nongenomic mechanisms involve rapid, transcription-independent actions of nuclear receptors. These processes occur within minutes and are crucial for swift cellular responses, such as ion channel modulation, cell migration, and acute stress responses.

##### Genomic mechanisms

Prior to binding to the specific DNA sequences of target genes known as hormone HREs to control transcription, NRs are typically in an inactive state and require activation through the binding of a cognate ligand.^15,137^ With many receptors, in the absence of ligands, molecular chaperone proteins such as heat shock protein 90 (HSP90) and HSP70 anchor them in the cytoplasm in an inactive state.^138^ Upon ligand binding, the NR undergoes a conformational shift that causes it to separate from the heat shock protein and become active.^139^ This allows the hormone–receptor complex to translocate into the cell nucleus after its nuclear localization sequence (NLS) has been revealed.^140,141^ Once inside, the complex binds to a specific sequence in the target gene known as an HRE to regulate its transcription. In addition to detaching the HSPs, ligand binding can promote receptor phosphorylation, which further enhances the ability of the receptor to bind to HREs.^142^ In general, NRs undergo a change from a non-DNA-binding form to a DNA-binding form upon activation, but some types, such as thyroid hormone receptors, retinoic acid receptors and retinoid X receptors, are retained in the nucleus regardless of their ligand binding status.^143^ They are already bound to HREs but complex with corepressor proteins, which makes them transcriptionally inactive.^144,145^ However, upon receiving ligand activation signals, the corepressor dissociates, and coactivator proteins are recruited. The NR/DNA complex then enlists more proteins, such as RNA polymerase, to aid in the transcription of DNA into mRNA.^146–148^

Nuclear receptors can directly bind DNA via three different modes, namely, homodimers, heterodimers, or monomers, depending on the type of NR.^13^ For example, glucocorticoid receptors (GRs) bind to HREs after forming homodimers.^149,150^ In contrast, retinoic acid receptor-related orphan receptor gamma (RORγ) binds as a monomer to the DNA response region to control gene transcription and expression.^151,152^ The retinoid X receptor (RXR) can either bind to itself as a homodimer or pair with other nuclear receptors as a heterodimer to activate downstream gene transcription.^153–155^ To make this process even more complicated, some NRs can form nonclassical heterodimers, which are only activated by outside ligands. Thus, unanticipated adverse effects could appear when developing therapeutics because NRs can operate through nonclassical routes.^156–159^

The identification of coregulatory proteins that interact with and control the transcriptional action of NRs in the mid-1990s was another pivotal development in our knowledge of the mechanism of action of NRs.^160,161^ These coregulators have a variety of functions, such as chromatin remodeling, which modifies the target gene’s transcriptional accessibility, or bridging, which maintains the binding of other coregulatory proteins.^162^ The intrinsic histone acetyltransferase (HAT) activity of coactivator proteins can promote gene transcription by reducing the affinity of histones for DNA.^163^ On the other hand, the recruitment of histone deacetylases (HDACs) by corepressor proteins increases the number of bonds between histones and DNA and suppresses gene transcription.^164^ These coactivators or corepressors enable nuclear receptors to function as inducible scaffolds that coordinate large transcriptional complexes, allowing different ligands to induce distinct receptor conformations.^163,165^ These changes could expose specific protein–protein interaction surfaces, making them available for coregulator binding. This hypothesis provides an explanation for how structurally different ligands, such as selective estrogen receptor modulators, could influence multiple genes in the same cell via the same receptor or how they could jointly regulate the expression of a single gene. Depending on the tissue-specific location of coregulators, the same ligand can also control specific genes in different tissues or at different developmental stages.^166,167^ Nuclear receptor/coregulator complexes that alter chromatin shape and DNA accessibility are produced by receptor-bound coregulators, which can also directly interact with the core RNA polymerase through their enzyme action. In addition, there is no set link between two distinct nuclear receptors, as they can change depending on the target genes and tissues that are engaged. Transcriptional regulation by NRs can be affected by epigenetic variables such as chromatin remodeling, DNA methylation and histone modification.^168,169^

##### Nongenomic mechanisms

**Fig. 6:**
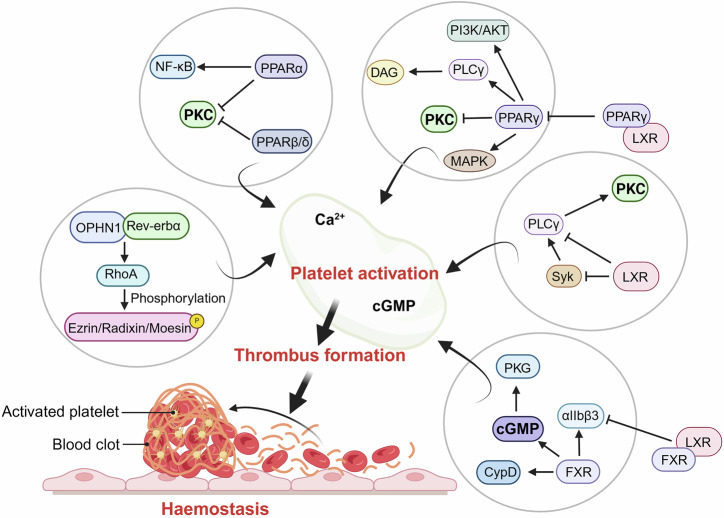
Platelets are good models for investigating the possible nongenomic impacts of nuclear receptors. Because of their anucleate origin, well-characterized mechanisms, and quick reactions, platelets offer a model system for examining any non-genomic impacts of the NRs. Non-genomic roles of NRs include controlling intracellular calcium levels, ion channel function, kinase and phosphatase activity, and second messenger synthesis. It has been discovered that human platelets contain a number of NRs, and that certain NR agonists have anti-platelet actions through a range of mechanisms

In addition to acting as transcription factors in the nucleus to regulate target gene expression (genomic function), NRs can also operate through a molecular mechanism independent of transcriptional activity (nongenomic function)^170–172^ (Fig. 5, Fig. 6). This nongenomic role requires NRs to be localized to specific subcellular structures,^173–175^ where they directly interact with various intracellular proteins to rapidly regulate cellular stress responses and signal transduction pathways. Their subcellular translocation can occur either by alternative splicing or posttranslational modifications through free shuttling or with the assistance of other proteins.^176,177^ As an example, the plasma membrane is home to a truncated variant of the thyroid hormone receptor. This localization enables them to participate in several biological processes, including the endoplasmic reticulum stress response, apoptosis, autophagy, fatty acid oxidation within mitochondria and the regulation of centrosome homeostasis and cell mitosis. Nongenomic NR signaling frequently involves phosphorylation cascades mediated through pathways associated with AMPK, cAMP/PKA, PI3K/AKT, and Hippo/Yap and signaling via Wnt/β-catenin and MAPK.^178–183^

Several NRs have been found to propagate rapid nongenomic effects for cellular protection in addition to their defined genomic effects. Their nongenomic functions operate through the control of kinase/phosphatase activity,^184,185^ intracellular calcium levels,^186,187^ ion channel function^188,189^ and the production of second messengers.^190^ Accumulating data indicate that RXRα also possesses extranuclear nongenomic functions. Previous studies have demonstrated that RXRα engages in some nongenomic activities through the creation of heterodimers with Nur77.^191^ In response to specific apoptotic stimuli, RXRα-Nur77 heterodimers are translocated from the nucleus to the mitochondria, where they regulate apoptosis.^192^ Recent research has shown that truncated RXRα (tRXRα) has an important function in the PI3K/AKT signaling pathway, where it interacts with the p85α subunit to increase AKT activation and promote cell proliferation both in vitro and in vivo.^193,194^ Notably, glucocorticoids (GCs) manifested nearly instantaneous nongenomic effects through nonspecific interactions with cell membranes and targeted interactions with cytosolic GRs (cGRs) or membrane-bound GRs (mGRs).^124,195,196^ This rapid nongenomic action of GCs could also contribute to their anti-inflammatory effects through prolonged genomic processes, particularly in managing inflammatory diseases (Fig. 6).^197,198^ For example, recent studies revealed that GCs quickly enhanced the effects of bronchodilators used to treat allergic asthma.^199^ Other NRs, including PPARα, LXR and FXR, have also been reported to perform nongenomic functions.^189,191,200^ The possibility of turning NR ligands into effective medicines should be explored through the characterization of these extragenomic processes and the discovery of new binding partners and pathways in addition to their known genomic effects.

Platelets are useful models for investigating the potential nongenomic effects of NRs because they lack nuclei and possess well-characterized reactions.^189,201^ ERβ, but not ERα, is known to be expressed on human platelets and to affect their function.^202^ In addition, some studies have shown that platelets isolated from male rats exhibit a stronger aggregation response than those from female rats do, likely due to an increase in androgenic steroids.^203,204^ This conclusion was supported by findings that castration reduced platelet aggregation in male rats,^205^ an effect that was reversed with testosterone treatment.^206^ LXR and FXR ligands negatively regulate platelet function by inhibiting platelet signaling and the formation of procoagulant-coated platelets.^207–210^ For example, LXR ligands induce a procoagulant state in platelets, which is marked by the exposure of phosphatidylserine and α-granule contents on the surface of the platelet, coupled with mitochondrial membrane depolarization, reduced calcium mobilization and decreased affinity of integrin αIIbβ3, which ultimately inhibits platelet aggregation.^209,211^ Similarly, FXR ligands can cause platelet swelling and transformation into procoagulant-coated platelets, a process dependent on cyclophilin D activity.^212^ FXR ligands also increased cGMP levels, which increased PKG activity and VASP S239 phosphorylation, further suppressing platelet activation.^189^ Other studies have identified PPARα as a key mediator of the antiplatelet effects of statins and fenofibrate.^213^ Treating platelets with PPARα ligands, such as fenofibrate or statins (e.g., simvastatin), inhibited ADP-stimulated platelet activation by increasing intracellular cAMP levels through a PPARα-dependent mechanism.^214^ This role of PPARα was further confirmed by experiments showing that fenofibrate-induced inhibition of platelet activation and prolonged bleeding time were absent in PPARα-deficient mice^215^; the reversion of this inhibitory effect by the PPARα antagonist GW6471 further highlighted its dependence on PPARα.

Subsequent studies revealed the unexpected presence of retinoic acid receptors (RARs) in the cytosol of Sertoli cells, hepatic stellate cells and neurons.^216–218^ In Sertoli cells, RARα was found to be a substrate for small ubiquitin-like modifier-2 (SUMO-2), and its cytosolic or nuclear localization was influenced by a dynamic process of sumoylation and desumoylation.^219,220^ Additionally, in Sertoli and hepatic stellate cells, RARα is retained in the cytosol through interactions with the cytoplasmic adaptor for RAR and TR (CART1) protein or with cytoskeletal proteins that sequester the receptor outside the nucleus.^144^ Furthermore, in hippocampal neurons, independent studies have shown that RARα is exported to dendritic RNA granules, where it is associated with a specific subset of mRNAs and RNA-binding proteins.^221–223^ Interestingly, in response to retinoic acid (RA), this extranuclear pool of RARα rapidly initiates the local translation of the postsynaptic glutamate receptor GluR1, leading to an increase in synaptic strength.^224,225^ In light of this growing body of knowledge, understanding these nongenomic mechanisms can lead to the development of new therapeutic strategies that exploit the rapid and diverse roles of nuclear receptors outside of their traditional genomic functions. By targeting the nongenomic pathways of nuclear receptors, it may be possible to create drugs with faster onset times and more specific effects, thereby increasing the efficacy of treatments for various diseases.

### Functions in physiology and pathology

Nuclear receptors are of key importance in physiology and pathology because they regulate multiple cellular functions (Fig. 5). For example, NHR-6, the only NR4A-type nuclear receptor in the roundworm *Caenorhabditis elegans*, has been implicated in the development of the nervous system.^226^ During development, the nervous system produces highly specialized neurons with distinct functional characteristics. A prime example is the *C. elegans* BAG chemosensory neurons.^227^ Under certain circumstances, BAG neurons sense the concentration of carbon dioxide in respiratory gas and translate it into reactions of attraction or repulsion; ETS-5 and its target gene NHR-6 are essential for this process.^228^ In contrast to ETS-5, which is engaged in both the attraction and avoidance of carbon dioxide, NHR-6 is an NR that is exclusively involved in the attraction process.^229^ These findings imply that gene regulatory mechanisms supported by NR subtypes have undergone evolutionary conservation. NHR-6 is necessary for BAGs to exhibit exceptional capacity to adaptively assign positive or negative valences to chemosensory stimuli, and it is a key modulator of the functional plasticity of the neural system.^226^ It is clear that NRs are of the greatest importance for the proper functioning of numerous processes in cellular lipid metabolism,^230,231^ energy homeostasis^232^ and the inflammatory response^233^; cell proliferation and differentiation are critically dependent on nuclear receptors.

Pathological changes in nuclear receptors, such as mutations, altered expression levels, or dysregulated activity, are linked to a wide range of diseases, including cancer, metabolic disorders, inflammation, and cardiovascular conditions. For example, the overexpression of ER in breast cancer drives tumor growth,^234^ whereas aberrant AR can cause therapy resistance in prostate cancer.^235^ Impaired PPAR function is associated with metabolic disorders such as obesity and type 2 diabetes,^236^ whereas dysregulated liver X receptors (LXRs) disrupt cholesterol homeostasis, exacerbating cardiovascular disease.^237^ Aberrant GR activity exacerbates inflammatory conditions and may induce corticosteroid resistance. Altered retinoid X receptor (RXR) function has been linked to neurodegenerative diseases such as Alzheimer’s disease. These pathological changes not only hold diagnostic and prognostic value but also serve as therapeutic targets. For example, ER, PR, and HER2 expression levels guide breast cancer classification and treatment, with SERMs such as tamoxifen proving effective in ER-positive patients. AR inhibitors are crucial in prostate cancer, where AR expression levels and mutations influence therapeutic outcomes. Additionally, radiolabeled ligands such as [F-18]-estradiol enable imaging of ER-positive tumors via PET scans,^238^ aiding in diagnosis and treatment monitoring. In summary, nuclear receptor alterations play a dual role in clinical practice as both therapeutic targets and biomarkers, underscoring their critical contribution to precision medicine and improved patient outcomes.

#### Cell fate

Nuclear receptors are essential for regulating cell fate through their function as transcription factors with effects on proliferation,^239^ differentiation,^240^ senescence^241^ and apoptosis.^242^ Through nongenomic mechanisms, NRs modulate signaling pathways that determine whether a cell will continue to divide, differentiate into a specific cell type, or undergo programmed cell death.^243^

The ER has been the most studied ER, mainly because of the estrogen dependence of the growth of certain breast cancer cells. The function of estrogen receptor alpha (ERα/ER) defines the most common type of breast cancer.^244,245^ Numerous associated elements that function as regulators of estrogen-driven transcriptional pathways have been discovered, indicating that the ER cannot function solely by itself.^246^ Enhancer sequences are cis-regulatory ER binding sites that are located mainly away from transcriptional start sites, according to genome-wide profiling of chromatin binding.^247^ There is growing evidence that global enhancer activation is involved in tumor aneuploidy and carcinogenesis.^248^ Short DNA enhancer elements are crucial for maintaining a coordinated gene expression program specific to cell types during development and differentiation.^249^ Other nuclear receptor ligands, such as all-trans retinoic acid (atRA) and 1,25(OH)2D3, induce cell differentiation.^250–252^ RA, the primary bioactive metabolite of retinol (vit A), has a range of pleiotropic effects on cell growth and differentiation, which are important in adult physiology and embryonic development.^253^ Members of the NR class of transactivators, specifically the retinoic acid receptors RARα, RARβ and RARγ, are the main mediators of activity.^254^ For example, prostate cancer-induced bone formation is decreased, and the endothelial-to-osteoblast transition (EC-to-OSB) is inhibited when the retinoic acid receptor (RAR) is activated.^255,256^ Treatment with the RARγ agonist palovarotene, which has been tested for heterotopic ossification in *fibrodysplasia ossificans progressiva*, inhibited osteoblast mineralization and the EC-to-OSB transition in vitro. It also reduces the growth and formation of tumor-induced bone in several models of osteogenic prostate cancer.^255^ Compared with mature hepatocytes, hepatocytes induced in a 3D environment exhibited lower activity of the nuclear receptor THRB. The addition of THRB ligands during differentiation upregulated not only the expression of CYP3A4 but also the expression of multiple hepatocyte-related genes and increased histone acetylation; the induced differentiated hepatocytes were more similar to mature hepatocytes.^21^ In addition, the nuclear receptor Nur77 regulates the expression of genes involved in fatty acid absorption and transport, thereby blocking the uptake of exogenous fatty acids by cells and inhibiting the signaling pathways that promote breast cancer cell proliferation.^27^ These findings highlight the significant role of NRs in controlling cell growth and differentiation.

NRs have also been shown to be involved in cellular senescence and cell death processes. Three members of the NR4A1/Nur77/NGFIB orphan nuclear hormone receptor subfamily (NR4A1, NR4A2 and NR4A3) have been the most studied. NR4A2 was reported to maintain anchorage-independent growth, thereby reducing cell death, known as anoikis, in HeLa cells.^257^ Following acute kidney injury (AKI), Nur77 and its family members Nurr1 and Nor-1 stimulate epithelial apoptosis. A conformational shift in Bcl2 and an increase in proapoptotic Bcl-xS protein levels are involved in Nur77-mediated kidney damage.^258^ Furthermore, YAP controls the transcription, phosphorylation and mitochondrial localization of NR4A1 to mediate the proapoptotic and antitumor actions of the Hippo pathway. In turn, NR4A1 acts as a feedback inhibitor of YAP, leading to its destruction, which prevents YAP from participating in carcinogenesis and liver regeneration.^259^ In addition, Nur77 interacts with DNMT3b, increasing GLS1 promoter methylation and decreasing GLS1 expression and glutaminolysis, which leads to the induction of HSC senescence.^260^ Hepatocyte nuclear factor 4α (HNF4α, NR2A1), another strongly conserved NR superfamily member, suppresses prostate tumorigenesis by arresting the cell cycle at the G2/M phase and p21-driven cell senescence.^261^ NR subfamily 1 group I member 2 (NR1I2), also called PXR (pregnane-X receptor) or PAR (pregnane-activated receptor), has been implicated in blunting the expression of the proapoptotic genes TP53 and BAK1 (BCL2 antagonist/killer 1) to prevent apoptosis from toxic bile acids.^262^ The retinoid RXRα has been shown to suppress the production of ITPR2 and control calcium signaling via ITPR2 and the mitochondrial calcium uniporter (MCU). After reducing the degree of DNA damage, the overexpression of RXRα delays replicative senescence.^263^ ZBTB17, a zinc finger protein, was shown in another study to interact with RXRα. Notably, ZBTB17 knockdown initiates a series of events, including DNA damage, decreased mitochondrial membrane potential (MMP), RXRα-dependent intracellular calcium signaling and cellular senescence.^264^ Under metabolic and genotoxic stresses, LXR/CD38 activation promoted lysosomal cholesterol efflux and nicotinamide adenine dinucleotide (NAD^+^) depletion in macrophages, which caused cholesterol-induced macrophage senescence and neurodegeneration.^265^

#### Cellular metabolism

**Fig. 7:**
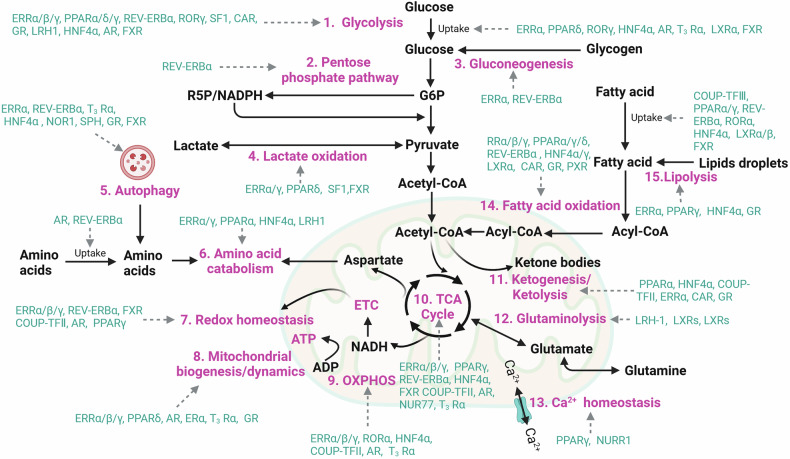
Examples of nuclear receptors involved in regulating metabolic pathways. Cellular metabolic pathways provide the energy and materials essential for cell survival. Nuclear receptors transcriptionally regulate genes that encode nutrient transporters and metabolic enzymes involved in these processes. The pathways include the following: 1. Glycolysis, 2. Pentose phosphate pathway (PPP), 3. Gluconeogenesis, 4. Lactate oxidation, 5. Autophagy, 6. Amino acid catabolism, 7. Redox homeostasis, 8. Mitochondrial biogenesis/dynamics, 9. Oxidative phosphorylation (OXPHOS), 10. Tricarboxylic acid (TCA) cycle, 11. Ketogenesis/ketolysis, 12. Glutaminolysis, 13. Ca^2+^ homeostasis, 14. Fatty acid oxidation (FAO), 15. Lipolysis

Members of the NR family control the pace at which different metabolic enzymes are synthesized in different tissues,^266^ which affects processes including the metabolism of glucose, lipids and bile acids; adipocyte differentiation; and plasma lipoprotein modulation.^267,268^ By influencing the balance between the storage, utilization and production of these macromolecules, NRs ensure metabolic flexibility, allowing the organism to adapt to fluctuations in energy availability and demand (Fig. 7). Their regulatory functions are particularly critical during periods of fasting, stress, or increased physical activity, where their precise control is essential for maintaining overall metabolic health.

##### Glucose metabolism and gluconeogenesis

Glucose is the primary metabolic fuel for mammals and plays a crucial role in maintaining energy homeostasis.^269,270^ During the fed state, circulating glucose originates primarily from the absorption of nutrients in the intestines.^271^ However, under fasting or energy-demanding conditions, the body relies on two additional sources to maintain glucose levels: the breakdown of stored glycogen through glycogenolysis and the production of new glucose molecules via gluconeogenesis.^272–274^ These complex metabolic processes are intricately regulated, and NRs play a key role in coordinating them. As molecular sensors, NRs detect changes in the body’s energy status and modulate the expression of genes involved in glucose metabolism.^275,276^ The activation of PXR by its ligand, pregnenolone-16α-carbonitrile (PCN), impaired glucose tolerance by dysregulating glucose transporter 2 (GLUT2) function through two distinct mechanisms.^277^ First, it reduced the expression of GLUT2 in both mouse liver and wild-type hepatocytes. Second, it triggers the transport of GLUT2 from the plasma membrane to the cytosol in the liver, thereby suppressing glucose uptake in primary hepatocytes. Additionally, data mining of published chromatin immunoprecipitation/sequencing results revealed that the *Glut2* gene is directly targeted by PXR. These findings may explain the diabetogenic effects of certain medications and environmental contaminants, positioning PXR in a potential novel diabetogenic pathway. At physiological concentrations found in the liver, glucose binds to and activates the transcriptional activity of LXRs, inducing the expression of LXR target genes with similar efficacy to that of oxysterols, which are well-known LXR ligands. Cholesterol homeostasis genes, which depend on LXR for their expression, were upregulated in the liver and intestines of fasted mice after refeeding with a glucose-rich diet,^278^ suggesting that glucose acted as an endogenous ligand for LXR.

Nuclear orphan receptor subfamily 4 group A member 1 (NR4A1), an important regulator of hepatic glucose homeostasis, was found to interact with the nuclear glycerol kinase Gyk during hepatic gluconeogenesis in the unfed state and in diabetes. Gyk acts as a corepressor of NR4A1, which attenuates the expression of target genes involved in hepatic gluconeogenesis and obstructs blood glucose regulation.^279^ Nuclear receptor subfamily 5 group A member 2 (NR5A2 or LRH1) and steroidogenic factor 1 (SF1 or NR5A1) work synergistically as key regulators of glucose-sensing systems in hepatic and steroidogenic tissues.^280^ These receptors are essential for the normal processing of glucose after eating through direct control of GCK and HK1 transcription.^281^ FXR combines signals from PKA and FOXA2 for hepatic glucose metabolism and production through two regulatory arms.^282^ The first is the phosphorylation of FXR by protein kinase A, which induces glucagon, agonist-activated FXR and CREB to combine and activate gluconeogenic genes. The physical interaction between FXR and the glucagon-activated FOXA2 transcription factor constitutes the second arm, which prevents FXR from stimulating the anti-gluconeogenic nuclear receptor SHP. Foxa2 knockdown did not influence the expression of glucagon-induced or FXR agonist-boosted gluconeogenic genes, suggesting that distinct subsets of FXR-sensitive genes are controlled by the PKA and FOXA2 pathways.^282^

NR coregulators play crucial roles in the regulation of glucose metabolism. For example, NR corepressors (NCoRs) operate within multiprotein complexes containing histone deacetylase 3 (HDAC3) to inhibit transcription, primarily through repressive chromatin remodeling at target loci. This process ultimately drives glucocorticoid receptor-dependent activation of hepatic gluconeogenesis. In hepatocytes, the double knockout of both NCoR1 and NCoR2 mimicked the hepatomegaly and fatty liver phenotype observed in HDAC3 knockouts by preventing glucocorticoid receptor binding.^283^ Similarly, nuclear receptor coactivator 3 (NCOA3) regulates the transcription of tyrosine-protein kinase (Fyn) in a PPARγ-dependent manner. NCOA3 deficiency was shown to worsen diabetic kidney disease by exacerbating albuminuria, glomerular sclerosis, and podocyte injury and impairing autophagy.^284^

##### Bile acid, sterol and lipid metabolism

Increasing evidence suggests that NRs play critical roles not only in glucose metabolism but also in the regulation of sterol, bile acid and lipid metabolism.^285–287^ The three ERR isoforms, as well as PPARα and PPARδ, are the main transactivators of genes involved in mitochondrial bioenergetics and fatty acid oxidation (FAO) and are viewed as primary controllers of mitochondrial FAO.^288,289^ For example, in addition to being a regulator of mitochondrial function, ERRα was established as a major transcriptional regulator of lipid biosynthesis and a promoter of nonalcoholic fatty liver disease (NAFLD/NASH).^290^ Medium-chain specific acyl-CoA dehydrogenase (*ACADM*), the first-discovered target gene of ERRα, encodes the initial rate-limiting enzyme in mitochondrial FAO; the promoter is also targeted by PPARα.^291^ In addition, a muscle-specific protein induced by PGC-1 and ERRs, PERM1 (PGC-1/ERR-induced regulator in muscle-1), promoted mitochondrial biogenesis and metabolism in cardiomyocytes. In this study, PERM1 was shown to interact with the proximal regions of PPAR response elements (PPREs) in the endogenous promoters of genes involved in fatty acid oxidation, and further results revealed that PERM1 interacted with PPRE to promote transcription, partly in a PPARα- and PGC-1α-dependent manner.^292^

PPARα is also a key regulator of fatty acid oxidation and can induce spontaneous fatty liver and hyperlipidemia in mice fed a standard diet. For example, during the early night, fasting mice exhibited acute PPARα-dependent hepatocyte activity, accompanied by increased circulating free fatty acids that could be further stimulated by adipocyte lipolysis. However, fasting resulted in mild hypoglycemia and hypothermia in PPARα^hep−/−^ mice, suggesting a role of PPARα activity in nonhepatic tissues.^293^ Liver-specific inactivation of Vps15, the essential regulatory subunit of class 3 PI3K, elicited mitochondrial depletion and failure to oxidize fatty acids through blunting of the transcriptional activity of PPARα. Vps15 deficiency led to the accumulation of the PPARα repressors HDAC3 and nuclear receptor corepressor 1 (NCoR1) in the liver due to disrupted autophagy. Activation of PPARα or inhibition of HDAC3 restored mitochondrial biogenesis and FAO in Vps15-deficient hepatocytes, revealing functions for class 3 PI3K and autophagy in the transcriptional coordination of mitochondrial metabolism.^294^ In addition, hepatic Ncor1 deletion in mice retarded atherosclerosis development by reprogramming bile acid metabolism and enhancing fecal cholesterol excretion, resulting in reduced plasma cholesterol levels and decreased hepatic cholesterol content in liver-specific Ncor1 knockout mice compared with those in controls fed an atherogenic diet for twelve weeks.^295^

NR4A1 was identified as a previously unrecognized constitutive regulator of adipocyte progenitor (AP) quiescence.^296^ In ex vivo experiments, NR4A1 gain-of-function reduced adipogenesis, whereas its loss-of-function increased adipogenesis.^296^ Compared with control mice, NR4A1 knockout mice fed a high-fat diet were more prone to obesity, with increased gene expression of PPARγ and FAS. Conversely, NR4A1 overexpression in 3T3-L1 preadipocytes inhibited adipogenesis. NR4A1 upregulated GATA binding protein 2 (GATA2), which in turn inhibited PPARγ. NR4A1 also suppressed sterol regulatory element-binding protein 1 (SREBP1) and its downstream target fatty acid synthase (FAS) by upregulating p53. Overall, NR4A1 inhibits adipocyte differentiation and lipid accumulation by increasing the expression of GATA2 and p53.^297^ Nuclear receptor subfamily 0 group B member 2 (NR0B2, also called SHP) is expressed at high levels in the liver and intestine, and its transcriptional activity can be increased by fibroblast growth factor 19 (human FGF19, mouse FGF15) signaling. FGF19 and SHP were observed to inhibit SREBF2 (sterol regulatory element binding transcription factor 2), which led to a reduction in intestinal NPC1L1 expression, cholesterol absorption and hypercholesterolemia.^298^ Another member of the nuclear receptor family, NR1A1, was reported to influence hepatic autophagy, lipid metabolism and adipocyte homeostasis by interacting with MED1 through a bridge formed by PGC1α.^299,300^

The expression of RORα/β/γ and REV-ERBα/β in the liver follows circadian rhythms. The liver genome contains high-order transcriptionally repressive hubs where REV-ERBα condensates are found, and these hubs are strongly associated with circadian gene repression.^301–303^ In mouse models, RORs bind to ROREs at night, whereas REV-ERBα/β bind to ROREs during the day to decrease lipogenesis. In the liver, REV-ERBα and REV-ERBβ work in concert, increasing the ability of HDAC3 and NCoR to control SREBP activity and maintain lipid homeostasis. They also affect cytochrome P4507A1 expression levels, which helps to maintain the equilibrium of bile acid metabolism.^304^ RORγ serves as a crucial activator of the entire cholesterol biosynthesis program, controlling SREBP2 by binding to genes involved in cholesterol biosynthesis and facilitating SREBP2 recruitment. At the loci of genes involved in cholesterol production, RORγ inhibition decreases chromatin acetylation and disrupts its connection with SREBP2. In immunocompetent animals and patient-derived xenografts, RORγ antagonists induce tumor regression.^305^

The regulation of bile acids, sterols and lipids is also associated with many other types of nuclear receptors. Through the regulation of Akr1b7 transcription, the nuclear receptor PXR effectively reduces AKI by increasing reactive oxygen species (ROS) generation, mitochondrial autophagy and mitochondrial dysfunction in lipid metabolism.^306^ It was recently demonstrated that the function of FXR in BA homeostasis depends on the phosphorylation of Tyr-67 by the FGF15/19 signaling-activated nonreceptor tyrosine kinase Src. According to Byun and colleagues, hepatic FXR phosphorylation by FGF15/19-induced Src preserves cholesterol homeostasis and protects against atherosclerosis.^307^ PXR activation upregulated intermediates in the Kandutsch–Russell cholesterol synthesis pathway in the liver and induced a number of cholesterol synthesis genes, including the rate-limiting HMRCR. In both mice and humans, PXR activation increased the level of plasma proprotein convertase subtilisin/kexin type 9 (PCSK9), a negative regulator of hepatic LDL absorption. A new regulator of PCSK9 and cholesterol synthesis, the PXR-SREBP2 pathway, also serves as a molecular mechanism for drug- and chemical-induced hypercholesterolemia.^308^

##### Mitochondria, OxPhos and the TCA cycle

Mitochondria serve as vital energetic and biosynthetic signaling hubs, taking in substrates from the cytoplasm to generate bioenergetic and biosynthetic building blocks.^309^ This process is achieved through the coordinated activity of several key metabolic pathways, including the electron transport chain (ETC), oxidative phosphorylation (OxPhos), the tricarboxylic acid (TCA) cycle, glycolysis, glutaminolysis and fatty acid oxidation (FAO). These pathways not only produce ATP but also provide intermediates essential for biosynthesis. Crucially, the function of mitochondria is tightly regulated by various nuclear receptors, which modulate gene expression to ensure proper mitochondrial activity and metabolic balance.

Several nuclear receptors are involved in regulating genes associated with the TCA cycle and OxPhos, including estrogen-related receptor alpha (ERRα), NUR77 (NR6A1) and PPARγ.^310–312^ One of the first major datasets used to highlight the role of ERRα in OxPhos regulation was a study that integrated PGC1α-induced GW transcriptional profiling with a software approach to identify cis-regulatory sequences.^313,314^ Mice lacking both ERRα and ERRγ presented the most extensive and profound disruption in skeletal muscle gene expression, with the ‘mitochondrial function’ pathway genes involved in OxPhos and the TCA cycle being the most significantly affected. Moreover, mice deficient in ERRβ and ERRγ exhibit impairments in lipid metabolism and branched-chain amino acid metabolism, specifically in the soleus muscle.^315^ Similarly, muscle-specific deletion of *Esrrg* (the gene encoding ERRγ) in mice reduces exercise capacity because of deficient mitochondrial activity.^316^ adipocytes lacking all ERR isoforms exhibited significant downregulation of genes involved in oxidative metabolic pathways, including those controlling the TCA cycle and OxPhos, leading to marked reductions in mitochondrial content and oxidative capacity.^317^ These results suggest that many mitochondrial actions are mediated through the stimulation of ERR expression and activity, underscoring the critical role of ERR in mitochondrial energy metabolism across various tissues.

The nuclear receptor Nur77 (also known as TR3 or NGFI-B), encoded by *Nr4a1*, belongs to the steroid/thyroid/retinoid superfamily and serves as a key regulator of energy metabolism. Nur77 is phosphorylated by ERK2 and translocated to the mitochondria upon glucose deprivation. Mitochondrial Nur77 binds to TPβ, a rate-limiting enzyme in FAO, to protect it from oxidation. This promoted the metabolic adaptation of melanoma cells by evading ROS-induced cell death.^318^ Ubiquitinated mitochondrial Nur77 interacts with the ubiquitin-binding domain of p62/SQSTM1 to form condensates capable of sequestering damaged mitochondria. An additional interaction between the N-terminal intrinsically disordered region (IDR) of Nur77 and the N-terminal PB1 domain of p62/SQSTM1 allows tethering of clustered mitochondria to the autophagy machinery, which endows Nur77-p62/SQSTM1 condensates with the magnitude and liquidity to act on the mitochondria.^319^ In response to the pathological accumulation of α-synuclein (α-syn) fibrils in Parkinson’s disease (PD), Nur77 is translocated from the cytoplasm to the mitochondria to improve PHB-mediated mitophagy by regulating c-Abl phosphorylation. Nur77 overexpression alleviated the expression of pS129-α-syn and protected dopamine (DA) neurons from the loss of α-syn in PD.^320^

NR1D1 (nuclear receptor subfamily 1 group D member 1) is the most highly upregulated nuclear receptor in abdominal aortic aneurysm (AAA) tissues. Knockout of Nr1d1 in vascular smooth muscle cells (VSMCs), but not in endothelial or myeloid cells, led to significant inhibition of AAA formation. Mechanistic investigations revealed that ACO2 (aconitase-2), a key enzyme in the TCA cycle, is a direct transcriptional target of NR1D1. By repressing ACO2, NR1D1 modulates mitochondrial metabolism. In the absence of NR1D1, ACO2 expression is restored, which subsequently corrects mitochondrial dysfunction during the early stages of Ang II infusion, even prior to the onset of AAA development.^321^

##### Catabolism of amino acids

Amino acid catabolism is vital during starvation to sustain glucose levels and provide alternative carbon sources, primarily in the liver but also in the kidneys, muscles and adipose tissue.^322^ This process involves transamination and oxidative deamination in the cytoplasm, producing metabolites such as α-ketoglutarate, pyruvate and acetyl-CoA, which feed into the TCA cycle.^323^ An essential NR in AA catabolism is ERRα, which is active in regulating genes involved in amino acid uptake and branched-chain amino acid metabolism.^324^ ERRα is also linked to reduced leucine oxidation under energy-demanding conditions.^325^ PPARα and HNF4α also regulate amino acid utilization. PPARα, in the presence of RXRα, inhibited HNF4α activity, suppressing serine dehydratase expression by promoting HNF4α degradation via the proteasome pathway. In PPARα-knockout mice, HNF4α levels and amino acid catabolizing enzyme (AACE) expression are elevated.^326^ In response to amino acid deficiency (AAD), MEF2D (myocyte enhancer factor 2D) and NR4A1 induce FAM134B2 expression and promote FAM134B2-mediated reticulophagy to maintain intracellular amino acid levels.^327^

#### Inflammation and immunity

Compelling evidence suggests that NRs can regulate inflammation and immunity, contributing to the pathogenesis of human diseases. For example, Nur77 binds to LPS, acting as an LPS receptor protein and playing a role in macrophage pyroptosis. Further research indicated that Nur77-LPS interactions activated NLRP3, resulting in the formation of an atypical inflammasome. These findings demonstrated that Nur77 promotes NLRP3 inflammasome activation, enhancing the host immune response to endotoxins.^174^ Similarly, Nur77 was identified as a key transcriptional regulator of the proinflammatory metabolic switch in macrophages. IDH expression was not downregulated in Nur77-deficient macrophages, which led to increased levels of succinate and other TCA cycle metabolites, independent of glutamine, resulting in increased nitric oxide and proinflammatory cytokine production in an SDH-dependent manner. Thus, Nur77 promotes an anti-inflammatory metabolic state in macrophages, offering protection against chronic inflammatory diseases such as atherosclerosis.^328^ Another nuclear receptor, RORα, regulates hepatic macrophage polarization and inflammation by inducing kruppel-like factor 4 (KLF4) expression. In myeloid-specific RORα-deficient animals, Kupffer cells (KCs) and bone marrow-derived macrophages fail to undergo M2 polarization, rendering them more susceptible to high-fat-diet-induced nonalcoholic steatohepatitis (NASH).^329^ Additionally, the orphan nuclear receptor TLX directly bound to the CD274 (PD-L1) gene promoter, showing a strong positive correlation with PD-L1 overexpression. Suppressing TLX significantly reduced the in vivo growth of glioma allografts and xenografts, preserved the antitumor immune response and markedly decreased the number of PD-L1-positive cells and glioma-associated macrophages.^330^ Nuclear receptor subfamily 1 group D member 1 (NR1D1) acts as a transcriptional repressor and plays a key role in regulating inflammation. The activation of NR1D1 decreased the expression of proinflammatory cytokines and matrix metalloproteinases (MMPs), whereas NR1D1 silencing had the opposite effect. Additionally, NR1D1 activation reduces ROS production and increases the generation of Nrf2-associated antioxidant enzymes.^331^

NRs also play crucial roles in modulating immune responses by directly regulating the function and activity of immune cells, such as influencing their development, differentiation and activation. For example, overexpression of NR4A1 inhibited the development of effector T cells through binding to the transcription factor AP-1 and inhibited its promotion of the expression of effector genes. NR4A1 binding encouraged the acetylation of histone 3 at lysine 27 (H3K27ac), which activated genes linked to tolerance. Deletion of NR4A1 increases immunity against tumors and chronic viruses, overcomes T-cell tolerance and enhances effector function.^332^ Similarly, another study revealed that NR4A triple knockout CAR-T lymphocytes presented traits and gene expression profiles typical of CD8^+^ effector T cells. The chromatin regions in these cells were enriched in binding motifs for transcription factors involved in T-cell activation, such as NF-κB and AP-1.^333^ These studies suggest that NR4A is a major regulator involved in the induction of T-cell dysfunction and that NR4A inhibition is a promising approach for cancer immunotherapy.

RARs and RA signaling play crucial roles in immune responses, influencing immune tolerance, tissue homing, lymph node formation and protective immunity.^334^ RARs include RARα, RARβ and RARγ, which are encoded by different genes with unique transcriptional variants and isoforms. In the resting state, RARα binds to transcriptional repressors to inhibit gene expression. However, upon binding to RA, RARα tends to bind to transcriptional activators, promoting gene expression^334^ RAR signaling has been shown to regulate immunity. For example, following TCR stimulation, extranuclear RARα is rapidly phosphorylated and recruited to the TCR signalosome. RA disrupted extranuclear RARα signaling, leading to reduced TCR activation and increased conversion of FOXP3+ regulatory T cells. RA is translocated to the nucleus by CRABP2, which is upregulated by TCR activation. Deletion of Crabp2 resulted in increased cytoplasmic RA, disrupting signalosome-associated RARα and consequently weakening autoimmune responses while diminishing antipathogen immunity.^335^

### Temporal expression changes

The expression of nuclear receptors is not only related to tissue, sex, and disease but also varies across different age groups within the same individual.^336^ These variations reflect developmental needs, metabolic changes, hormonal shifts, and adaptations to aging-related physiological demands. Generally, developmental NRs such as RARs, PPARs and RXRs are highly expressed during embryogenesis and early development to support rapid growth, differentiation, and development.^337^ During adolescence, ARs, ERα and ERβ are elevated to correlate with puberty and the onset of secondary sexual characteristics, whereas GRs act to moderate stress responses and energy metabolism during periods of rapid physical and emotional development. PPARs, LXRs, and FXRs live in adult individuals to maintain lipid and glucose homeostasis, supporting metabolic stability during peak years of physical activity and reproductive capability. Diet, exercise, and stress significantly modulate NR expression, affecting metabolic and endocrine balance during this period. The expression of these NRs decreases with increasing age, which causes impaired lipid metabolism and insulin sensitivity, cholesterol and bile acid metabolism, and calcium homeostasis and immune function, leading to an increased risk of metabolic syndrome, compromised organ function and chronic inflammation. For example, Nr4a1 is reduced in peripheral blood mononuclear cells and CA1 pyramidal neurons with aging, which impairs cognition and excitatory synaptic function.^338^ Monitoring age-specific NR expression patterns could guide personalized therapies to optimize metabolic health and prevent age-related diseases.

### Degradation and metabolic cycling

The metabolic cycling process of NRs, including their synthesis, activation, degradation, and recycling, is integral to maintaining homeostasis and ensuring that NRs can adapt to changing physiological demands. Since the activation and transcriptional regulation of nuclear receptors have been discussed above, we mainly emphasize their degradation and recycling. The degradation of nuclear receptors is a highly regulated process that involves proteasomal and lysosomal pathways, ensuring the dynamic control of receptor levels in response to physiological and environmental cues. Ligand binding can either stabilize or destabilize NRs by inducing conformational changes that influence ubiquitination.^339,340^ In addition, posttranslational modifications (PTMs), such as phosphorylation,^341^ SUMOylation^342^ and acetylation,^343^ modify NR degradation rates by altering their interaction with ubiquitin ligases or stabilizing proteins. Moreover, many NRs, such as REV-ERBs and PPARs, exhibit circadian cycling, with their expression and activity oscillating in a 24-hour rhythm to align with metabolic needs.^344,345^ These rhythms are coregulated by core circadian clock genes (e.g., CLOCK and BMAL1), which synchronize metabolic functions with environmental cycles such as light and feeding. Altogether, cycling of NRs ensures appropriate receptor levels, preventing excessive or prolonged signaling that could lead to cellular dysfunction.

### Alterations cause by aging

With aging, the efficiency of key regulatory pathways declines, leading to altered NR turnover, disrupted signaling, and increased susceptibility to age-related diseases.^346^ Proteasomal dysfunction, a hallmark of aging, impairs the degradation of damaged or misfolded proteins, including NRs. This results in the accumulation of inactive or malfunctioning receptors, which disrupt signaling and contribute to metabolic and endocrine dysregulation, key features of aging. These disruptions play a central role in the development of diseases such as obesity, diabetes, cardiovascular conditions, and neurodegenerative disorders.^347,348^ Additionally, aging reduces autophagic flux, hindering the clearance of damaged proteins, including aggregated NRs. As autophagy becomes less efficient with age, NR aggregates persist, disrupting receptor signaling and promoting chronic inflammation, mitochondrial dysfunction, and cellular stress.^349,350^ These factors are implicated in the progression of age-related diseases like Alzheimer’s, diabetes, and frailty. Oxidative stress also contributes to aging-related cellular damage by directly modifying NRs. ROS cause conformational changes in NRs, preventing their proper ubiquitination and degradation. This oxidation makes NRs resistant to degradation, leading to the accumulation of damaged receptors and further dysfunction in signaling pathways.^351^ Understanding these age-related changes offers valuable insights for developing targeted therapies to restore NR homeostasis and mitigate aging-associated disorders.

## Targeting nuclear receptors for disease management

Given the critical role of NRs in physiological and pathological processes, they constitute a highly significant and privileged class of intracellular druggable targets for treating various diseases.^52,352–354^ By modulating these receptors, it is possible to influence a wide range of conditions, from metabolic disorders such as diabetes and obesity to immune-related diseases such as autoimmune disorders and inflammatory conditions (Tables 1, 2). NRs are central to cancer progression and hormone regulation, making them prime targets for oncology and hormone therapies. The ability to design drugs that specifically interact with nuclear receptors enhances their potential for precision medicine, offering more targeted treatments with fewer side effects across a broad spectrum of diseases.

**Table 1:** Summary of completed clinical trials of nuclear receptor-targeted drugs

Target	NCT Number	Results	Conditions	Agents	Phases
Retinoic acid receptor-α (RARα)	NCT02273102	COMPLETED (No Results Posted)	Acute Myelogenous Leukemia; Myelodysplastic Syndromes; Leukemia	Tranylcypromine; Tretinoin	PHASE1
Retinoic acid receptor-γ (RARγ)	NCT02521792	TERMINATED (The study was stopped prematurely and due to the small number of evaluable subjects, there was insufficient data to conduct any efficacy analyses. Consequently, no summary statistics are available for any of the secondary outcome measures)	Fibrodysplasia Ossificans Progressiva	Palovarotene	PHASE2
	NCT02190747	COMPLETED (Supporting further evaluation of palovarotene for prevention of HO in FOP in larger studies^491^)	Fibrodysplasia Ossificans Progressiva (FOP)	Palovarotene; Placebo	PHASE2
	NCT02279095	COMPLETED (RAR-γ agonists are potent inhibitors of heterotopic ossification in mouse models^492^)	Fibrodysplasia Ossificans Progressiva	Palovarotene	PHASE2
Peroxisome proliferator-activated receptor-α (PPARα)	NCT00809068	COMPLETED (No Results Posted)	HDL Cholesterol	Fenofibrate and tibolone; Tibolone	PHASE4
	NCT03662984	COMPLETED (No Results Posted)	Myocardial Insulin Sensitivity; Impaired Glucose Metabolism; Diastolic Dysfunction	Ciprofibrate 100Mg Tablet; Placebo Oral Tablet	PHASE3
	NCT03345901	TERMINATED (No Reason Posted)	Diabetic Retinopathy; Diabetic Macular Edema	Pemafibrate; Placebo	PHASE3
	NCT03829436	COMPLETED (No Results Posted)	Hepatocellular Carcinoma; Metastatic Castration; Resistant Prostate Cancer; Renal Cell Carcinoma; Non-small Cell Lung Cancer;Colorectal Cancer;Squamous Cell Carcinoma of Head and Neck;Triple-Negative Breast Cancer; Urothelial Carcinoma; Cholangiocarcinoma; GastroEsophageal Cancer; Pancreatic Cancer; Sarcoma	Part 1 TPST-1120; Part 2 TPST-1120 + nivolumab; Part 3 TPST-1120; Part 4 TPST-1120 + nivolumab	PHASE1
	NCT00483210	WITHDRAWN (Study results have not been submitted. This may be because the study is not done, the deadline for submitting results has not passed, this study is not required to submit results, or the sponsor or investigator has requested or received a certification to delay submitting the results)	Tissue Lipid Metabolism	Fenofibrate	NA
Peroxisome proliferator-activated receptor-β/δ (PPAR-βδ)	NCT03040856	WITHDRAWN (Study results have not been submitted. This may be because the study is not done, the deadline for submitting results has not passed, this study is not required to submit results, or the sponsor or investigator has requested or received a certification to delay submitting the results)	Omega 3 Supplements During Pregnancy	Alpha Linolenic Acid; Omega 3 enriched diet (DHA + EPA); OTHER: Placebo	NA
Peroxisome proliferator-activated receptor-γ (PPAR-γ)	NCT03080480	TERMINATED (No Reason Posted)	Chronic Granulomatous Disease	Pioglitazone	PHASE1; PHASE2
	NCT00166803	SUSPENDED (No Reason Posted)	Diabetes Mellitus, Atherosclerosis	Rosiglitazone	NA
	NCT00408434	COMPLETED (Efatutazone demonstrates acceptable tolerability in patients with advanced malignancies)	Neoplasm	Efatutazone	PHASE1
	NCT02774343	COMPLETED (No conclusion)	Cocaine Use Disorder; Alcohol Use Disorder	Pioglitazone; Placebo;	PHASE1; PHASE2
	NCT00603941	TERMINATED (The study was halted prematurely due to low enrollment and failure to establish recommendation of Phase 2 dose. Analysis for the primary efficacy outcomes were also combined for the study)	Anaplastic Thyroid Cancer	CS-7017; Paclitaxel	PHASE1; PHASE2
	NCT00193648	COMPLETED (No Results Posted)	Focal Glomerulosclerosis	Rosiglitazone (Avandia); Adalimumab (Humira)	PHASE1
	NCT00407862	COMPLETED (No Results Posted)	Hypertension; Impaired Glucose Tolerance	Telmisartan 80 mg; Losartan 50 mg	PHASE4
	NCT06266598	COMPLETED (No Results Posted)	Childhood Obesity; Insulin Resistance	Metformin	PHASE4
	NCT03231033	COMPLETED (No Results Posted)	Autoimmune Pulmonary Alveolar Proteinosis	Pioglitazone	PHASE1
	NCT00285805	COMPLETED (No Results Posted)	Insulin Resistance	Rosiglitazone versus placebo	NA
	NCT01068444	COMPLETED (No Results Posted)	Hepatitis	Pioglitazone; placebo	PHASE2
	NCT02694874	COMPLETED (No Results Posted)	Malaria	Rosiglitazone; Placebo	NA
	NCT01395784	COMPLETED (No conclusion)	Opioid Abuse	Pioglitazone	PHASE2
	NCT01019356	COMPLETED (No Results Posted)	Polycystic Ovary Syndrome	Rosiglitazone; Acarbose	NA
	NCT02696577	COMPLETED (No Results Posted)	Intrauterine Growth Restriction	Low dose aspirin; Omega 3	PHASE2
	NCT00452192	COMPLETED (No Results Posted)	Metabolic Syndrome	Telmisartan	PHASE3
	NCT00868140	TERMINATED (No Reason Posted)	Polycystic Ovary Syndrome	Pioglitazone; Placebo	NA
	NCT03868566	TERMINATED (No Reason Posted)	NASH-Nonalcoholic Steatohepatitis	SNP-612 dose1; SNP-612 dose2	PHASE2
	NCT02444910	COMPLETED (No Results Posted)	Diabetes Mellitus, Type 2	KDT501	PHASE2
	NCT01589445	COMPLETED (No conclusion)	Type 2 Diabetes Mellitus	Pioglitazone hydrochloride; Metformin hydrochloride	PHASE4
	NCT03864523	COMPLETED (No Results Posted)	Adrenomyeloneuropathy; X-linked Adrenoleukodystrophy	Pioglitazone	PHASE2
Farnesoid X receptor (FXR)	NCT01492998	TERMINATED (No Reason Posted)	Chronic Hepatitis C	Guggulsterone	NA
	NCT02654236	COMPLETED (No conclusion)	Alcohol Consumption	Placebo; 10 mg Obeticholic Acid (OCA)	NA
	NCT03272009	COMPLETED (No Results Posted)	Hepatitis B, Chronic	EYP001a; Placebo;	PHASE1
				Entecavir; peg-interferon alfa-2a	
	NCT01625026	COMPLETED (No Results Posted)	Obesity; Gallstones	Obeticholic acid; Placebo	PHASE2
	NCT01265498	COMPLETED (No conclusion)	Nonalcoholic Fatty Liver Disease (NAFLD); Nonalcoholic Steatohepatitis (NASH)	Obeticholic acid; placebo	PHASE2
	NCT01585025	COMPLETED (No conclusion)	Primary Bile Acid Malabsorption; Secondary Bile Acid Malabsorption; Chronic Diarrhea	Obeticholic acid	PHASE2
	NCT04365933	COMPLETED (No Results Posted)	Hepatitis B, Chronic	EYP001a; Entecavir; Pegylated interferon alpha2a	PHASE2
	NCT04465916	TERMINATED (No Reason Posted)	Hepatitis B, Chronic	EYP001a; Placebo; Nucleotide analog (Entecavir or Tenofovir Disoproxil)	PHASE2
	NCT02430077	COMPLETED (No conclusion)	Familial Partial Lipodystrophy	Obeticholic Acid; Placebo	PHASE2
	NCT00465751	COMPLETED (No Results Posted)	Metabolic Syndrome; Familial Hypertriglyceridemia; Familial Combined Hyperlipidemia	Chenodeoxycholic acid; Placebo capsules	EARLY_PHASE1
	NCT03320616	COMPLETED (No Results Posted)	Hepatitis B, Chronic	EYP001a	PHASE1
	NCT05450887	COMPLETED (No Results Posted)	Primary Biliary Cholangitis	Obeticholic Acid Tablets; UDCA; Placebo	PHASE3
	NCT02855164	TERMINATED (No outputs were planned; and are not available for determining the effects of tropifexor on primary endpoints in the subset of patients who had historical biopsy data)	NASH	Tropifexor (LJN452); Placebo	PHASE2
	NCT01999101	COMPLETED (No Results Posted)	NAFLD	Px-104	PHASE2
	NCT05321524	TERMINATED (No Reason Posted)	Biliary Atresia	OCA 0.1 mg; OCA 1.5 mg; OCA 5 mg	PHASE2
	NCT05203367	WITHDRAWN (Study results have not been submitted. This may be because the study is not done, the deadline for submitting results has not passed, this study is not required to submit results, or the sponsor or investigator has requested or received a certification to delay submitting the results)	NAFLD	BAR502; Placebo	PHASE1
	NCT03469583	COMPLETED (No conclusion)	Hepatitis B, Chronic	EYP001; Entecavir 1 MG	PHASE1
	NCT05591079	COMPLETED (No Results Posted)	NASH	CS0159 (Linafexor)	PHASE2
	NCT05604287	TERMINATED (No Reason Posted)	Healthy Participants	ID119031166M; Placebo	PHASE1
	NCT04408937	COMPLETED (No Results Posted)	Liver Disease	Triopifexor; Placebo	PHASE1
	NCT02158351	COMPLETED (No Results Posted)	Simple Steatosis (SS); Obesity; NASH	PROCEDURE: liver and white adipose tissue biopsies	NA
	NCT02177136	COMPLETED (Treatment with OCA 5-10 mg reduced serum alkaline phosphatase in patients with PSC^493^)	Primary Sclerosing Cholangitis (PSC)	Obeticholic Acid (OCA); Placebo	PHASE2
	NCT02713243	COMPLETED (Tropifexor had acceptable safety and tolerability and exhibited therapeutic potential^494^)	Primary Bile Acid Diarrhea	LJN452; Placebo to LJN452	PHASE2
	NCT04328077	COMPLETED (No conclusion)	NASH	TERN-101; Placebo	PHASE2
	NCT03270527	COMPLETED (No Results Posted)	Lipids; Lipid Metabolism; Healthy	High SFA diet (Diet 1); Low SFA diet (Diet 2)	NA
	NCT03598920	COMPLETED (No Results Posted)	Obesity, Morbid	Asspire Assist; Nutritional consulting	NA
	NCT05415722	COMPLETED (No Results Posted)	NASH	TERN-501; TERN-101; Placebo	PHASE2
	NCT00550862	TERMINATED (No Reason Posted)	Liver Cirrhosis, Biliary	INT-747; Ursodeoxycholic Acid (URSO); Placebo	PHASE2
	NCT02308111	TERMINATED (The estimated effect of treatment from the ITT population was underpowered and potentially biased, resulting in difficulties in the interpretation of the tests of hypotheses for the primary and key secondary endpoints)	Liver Cirrhosis, Biliary	Obeticholic Acid (OCA); Placebo	PHASE4
	NCT00501592	COMPLETED (25 or 50 mg OCA s was well tolerated, increased insulin sensitivity, and reduced markers of liver inflammation and fibrosis^495^)	Diabetes Mellitus, Type II; Fatty Liver	INT-747; Placebo	PHASE2
	NCT03836937	COMPLETED (No Results Posted)	NAFLD	Obeticholic acid	NA
	NCT02430077	COMPLETED (No Results Posted)	Familial Partial Lipodystrophy	Obeticholic Acid; Placebo	PHASE2
	NCT05415722	COMPLETED (No Results Posted)	NASH	TERN-501; TERN-101; Placebo	PHASE2
Vitamin D receptor (VDR)	NCT04847947	COMPLETED (No Results Posted)	Prefrail Elderly	Cholecalciferol; Placebo	PHASE3
	NCT00677534	COMPLETED (No Results Posted)	End-Stage Renal Disease	Cholecalciferol	NA
	NCT01265615	COMPLETED (No Results Posted)	Cardiorenal Syndrome; Chronic Allograft Nephropathy	Paricalcitol; Calcitriol; Cholecalciferol	PHASE4
	NCT03331562	COMPLETED (No Results Posted)	Pancreatic Cancer; Pancreas Adenocarcinoma; Advanced Pancreatic Cancer; Metastatic Pancreatic Cancer; Metastatic Pancreatic Adenocarcinoma	Pembrolizumab; paricalcitol; placebo	PHASE2
	NCT04140292	COMPLETED (Oral VD3 pretreatment significantly improves actinic keratoses clinical responses to PDT^496^)	Actinic Keratosis	Photodynamic therapy (PDT); Vitamin D3	PHASE2
	NCT00301067	COMPLETED (No Results Posted)	Metastatic Melanoma	DIETARY_SUPPLEMENT: Calcitriol; Temozolomide	PHASE1; PHASE2
	NCT00507442	COMPLETED (VDR and VCD-mod are preferred for clinical practice and further comparative testing^497^)	Multiple Myeloma	VELCADE (bortezomib); dexamethasone; cyclophosphamide; Revlimid (lenalidomide)	PHASE1; PHASE2
	NCT02931162	COMPLETED (No Results Posted)	Ulcerative Colitis	OTHER: Herb-partitioned moxibustion; OTHER: Sham herb-partitioned moxibustion	NA
	NCT01084538	COMPLETED (No Results Posted)	Parathyroid Hormone	Zemplar iv (paricalcitol iv)	NA
	NCT01092130	COMPLETED (Six weeks of supplementation with 2,000 IU VitD3 increased 25-hydroxyvitamin D3 levels and decreased PRA and plasma renin concentration^498^)	Chronic Heart Failure	Vitamin D	PHASE2
	NCT01447355	COMPLETED (No Results Posted)	Healthy, no Evidence of Disease; Skin Cancer	Cholecalciferol	PHASE1
	NCT02856503	WITHDRAWN (Study results have not been submitted. This may be because the study is not done, the deadline for submitting results has not passed, this study is not required to submit results, or the sponsor or investigator has requested or received a certification to delay submitting the results)	Breast Cancer; Ductal Carcinoma In situ	Vitamin D3	PHASE1; PHASE2
	NCT02282800	COMPLETED (No Results Posted)	Aggressive Periodontitis	GENETIC: Vitamin D receptor	
	NCT03602430	COMPLETED (Cholecalciferol was effective, tolerable, inexpensive pharmacotherapeutic option to overcome vitamin D deficiency^499^)	Hemodialysis Complication; Vitamin D Deficiency; Vascular Calcification	Cholecalciferol; Placebo	PHASE2; PHASE3
	NCT05753280	COMPLETED (No Results Posted)	Chronic Hepatitis b	Vitamin D	PHASE4
	NCT01222234	COMPLETED (No Results Posted)	Chronic Kidney Disease (CKD); End-stage Renal Disease (ESRD)	Cholecalciferol - CKD; DEVICE: Calcitriol - CKD; Cholecalciferol - non-CKD	NA
	NCT00585442	TERMINATED (No Reason Posted)	Hypertension; Vitamin D Deficiency	Calcitriol; Placebo	EARLY_PHASE1
	NCT00084864	TERMINATED (Study was activated prior to Roswell Park Cancer Institute (RPCI) putting a system in place to centralize data entry and data management. Data entry was done by PI’s staff. PI left institute and left incomplete data)	Prostate Cancer	DIETARY_SUPPLEMENT: calcitriol; dexamethasone; clinical observation	PHASE2
	NCT04197089	COMPLETED (No Results Posted)	Urothelial Carcinoma	Vitamin D	PHASE4
	NCT03415854	COMPLETED (No Results Posted)	Pancreatic Cancer; Pancreatic Ductal Adenocarcinoma; Pancreatic Adenocarcinoma; Pancreas Metastases; Adenocarcinoma	Paricalcitol (Zemplar)	PHASE2
	NCT00851552	TERMINATED (Due to the study’s early termination and inadequate number of patients, no patients were analyzed)	Lymphoma	Rituximab; bortezomib; pegylated liposomal doxorubicin hydrochloride;	PHASE2
	NCT02854163	COMPLETED (No Results Posted)	Psoriatic Arthritis	Secukinumab	PHASE2
	NCT02783924	COMPLETED (No Results Posted)	Vitamin D Deficiency	Cholecalciferol; Placebo	PHASE2
	NCT01580007	COMPLETED (Adjunct therapy with PBA+vitD3 or vitD3 or PBA to standard short-course therapy demonstrated beneficial effects toward clinical recovery^500^)	Pulmonary Tuberculosis	Active Sodium Phenylbutyrate and active cholecalciferol; Placebo Sodium Phenylbutyrate plus active cholecalciferol; Active Sodium Phenylbutyrate and placebo cholecalciferol; Placebo Sodium Phenylbutyrate plus placebo cholecalciferol	PHASE2
	NCT02527668	COMPLETED (No significant change in intramyonuclear VDR in response to either form of vitamin D vs. placebo; Type I FCSA significantly increased with VD3^501^)	Osteopenia/Osteoporosis	DIETARY_SUPPLEMENT: Calcifediol; Vitamin D3; Placebo	PHASE2
	NCT03857893	TERMINATED (No Reason Posted)	Vulvo-vaginal Atrophy; Genitourinary Syndrome of Menopause	DEVICE: Dynamic Quadripolar Radio-Frequency treatment; pH-Cream	NA
	NCT01640496	WITHDRAWN (Study results have not been submitted. This may be because the study is not done, the deadline for submitting results has not passed, this study is not required to submit results, or the sponsor or investigator has requested or received a certification to delay submitting the results)	Ulcerative Colitis; Inflammatory Bowel Disease	Vitamin D3; Placebo	NA
	NCT01264874	TERMINATED (No Reason Posted)	Melanoma	Vitamin D3 (Colecalciferol); placebo	PHASE3
	NCT01325311	COMPLETED (No Results Posted)	Prostate Adenocarcinoma; Stage I Prostate Cancer; Stage IIA Prostate Cancer; Stage IIB Prostate Cancer	Cholecalciferol; Genistein; Placebo	PHASE2
	NCT00491920	COMPLETED (No Results Posted)	Osteoporosis; Osteopenia	Cholecalciferol (Vitamin D3); placebo	PHASE4
	NCT01608451	TERMINATED (No Reason Posted)	Locally Advanced Breast Cancer and Large Operable Breast Cancer	Cholecalciferol; Inj. Progesterone	PHASE3
	NCT04368520	WITHDRAWN (Study results have not been submitted. This may be because the study is not done, the deadline for submitting results has not passed, this study is not required to submit results, or the sponsor or investigator has requested or received a certification to delay submitting the results)	Common Cold	DIETARY_SUPPLEMENT: Vitamin D3; Vitamin D3; Placebo	PHASE2
	NCT01996423	COMPLETED (Weekly VD supplementation improved VD status but did not modify AD severity or type 2 immunity biomarkers^502^)	Atopic Dermatitis	DIETARY_SUPPLEMENT: Vitamin D3; Placebo	NA
	NCT02802449	COMPLETED (No Results Posted)	Hypovitaminosis D	25 Hydroxy- Vitamin D3 [25 (OH) D3]; Placebo	NA
	NCT02856776	COMPLETED (No Results Posted)	Vitamin D Insufficiency	DIETARY_SUPPLEMENT: Vitamin D3	NA
	NCT01656070	COMPLETED (No Results Posted)	HIV Disease; Vitamin D Deficiency; Hypovitaminosis D; Hyperparathyroidism	Oral cholecalciferol 1000000 UI (vitamin D3); Placebo	PHASE2
	NCT00010231	COMPLETED (No Results Posted)	Prostate Cancer	DIETARY_SUPPLEMENT: calcitriol; dexamethasone	PHASE1
	NCT04443387	COMPLETED (No Results Posted)	Effect of Drug	Vitamin D; Placebo	NA
	NCT02714361	COMPLETED (No Results Posted)	Iron Deficiency; Anemia; Deficiency, Nutritional, With Poor Iron Absorption; Vitamin D Deficiency	DIETARY_SUPPLEMENT: Vitamin D3 supplement; Placebo	NA
	NCT01721915	COMPLETED (No Results Posted)	Polycystic Ovary Syndrome; Healthy; Vitamin D Deficiency	Vitamin D supplementation; Placebo	PHASE4
	NCT03919812	COMPLETED (No Results Posted)	Thalassemia; Tuberculosis; Vitamin D Deficiency	DIETARY_SUPPLEMENT: Cholecalciferol supplementation	NA
	NCT01304927	COMPLETED (High-dose vitamin D supplementation has beneficial effects on glucose homeostasis and HDL cholesterol levels in infertile men^503^)	Male Infertility	Cholecalciferol and calcium; Placebo	PHASE2; PHASE3
	NCT02805231	COMPLETED (No Results Posted)	Primary Open- Angle Glaucoma	Vitamin D receptor polymorphic analysis	NA
	NCT04593524	COMPLETED (No Results Posted)	Tuberculosis	DIETARY_SUPPLEMENT: vitamin D 1000 IU and A 6000 IU supplementation and nutritional counseling; nutritional Counseling	NA
	NCT01204528	COMPLETED (Decline Endothelial function in patients with moderate CKD in patients with moderate CKD^504^)	Chronic Kidney Disease	Zemplar	PHASE2; PHASE3
	NCT02276755	COMPLETED (Vitamin D supplementation did not result in a lower risk of tuberculosis infection, tuberculosis disease, or acute respiratory infection^505^)	Latent Tuberculosis	DIETARY_SUPPLEMENT: Cholecalciferol (vitamin D3); Placebo	PHASE3
	NCT01635062	COMPLETED (No conclusion)	Type 2 Diabetes; Obesity	Calcitriol; Placebo	NA
	NCT03028519	COMPLETED (No Results Posted)	Ovarian Cancer	Vitamin D3	EARLY_PHASE1
	NCT02018133	COMPLETED (No Results Posted)	CKD Stage 3/4	Vitamin D2 mg daily for 2 weeks oral paricalcitol;	NA
				Placebo	
	NCT05505253	COMPLETED (No Results Posted)	Pelvic Organ Prolapse	Alfacalcidol 0.0005 MG; Placebo	PHASE3
	NCT03021629	COMPLETED (No Results Posted)	Vitamin D Deficiency; Primary Open-angle Glaucoma; High Myopia; Vitamin D Receptor Polymorphisms	Serum levels of 1a, 25-Dihydroxyvitamin D3 were measured by an enzyme-linked immuno-absorbent assay	
	NCT00656032	COMPLETED (No Results Posted)	End Stage Renal Disease	Paricalcitol; cinacalcet	PHASE2
	NCT01436747	COMPLETED (No Results Posted)	Disorder of Transplanted Kidney; Proteinuria;Albuminuria	Paricalcitol; Placebo	PHASE3
	NCT00587158	COMPLETED (Addition of 2 μg/day paricalcitol lowers residual proteinuria in kidney transplant recipients^506^)	Transplant; Failure, Kidney; Renal Disease, End Stage; Hyperparathyroidism, Secondary	Paricalcitol; Corticosteroid Avoidance Immune Suppression Protocol	NA
	NCT00796679	COMPLETED (52 weeks of treatment with oral paricalcitol significantly improved secondary hyperparathyroidism^507^)	Chronic Kidney Disease	Paricalcitol	NA
	NCT05376865	COMPLETED (No Results Posted)	Overweight; Hypovitaminosis D	DIETARY_SUPPLEMENT: Vitamin D supplement	NA
	NCT00977080	COMPLETED (Paricalcitol versus cinacalcet plus low-dose vitamin D provided superior control of intact parathyroid hormone)	Chronic Kidney Disease; Secondary Hyperparathyroidism; Hemodialysis	Paricalcitol; Cinacalcet	PHASE4
	NCT01134315	TERMINATED (No Reason Posted)	Secondary Hyperparathyroidism; End-Stage Renal Disease	Paricalcitol; Calcitriol	
	NCT01393808	COMPLETED (Paricalcitol prevents a sodium-induced increase in albuminuria^508^)	Type 2 Diabetes	Paricalcitol; placebo	PHASE2
	NCT01197664	TERMINATED (No Reason Posted)	Mucinous Adenocarcinoma of the Rectum; Stage IIA Rectal Cancer; Stage IIB Rectal Cancer; Stage IIC Rectal Cancer; Stage IIIB Rectal Cancer; Stage IIIC Rectal Cancer	Paricalcitol; radiation therapy; fluorouracil	PHASE1
	NCT00294866	COMPLETED (No Results Posted)	Kidney Failure	Paricalcitol	PHASE4
	NCT00186901	COMPLETED (No Results Posted)	Leukemia, Lymphoblastic, Acute; Osteoporosis	Calcium carbonate (Tums), vitamin D; Placebo	PHASE3
	NCT00421733	COMPLETED (Addition of 2 μg/day paricalcitol to Addition of 2 μg/day paricalcitol to RAAS inhibition safely lowers residual albuminuria in patients with diabetic nephropathyinhibition safely lowers residual albuminuria in patients with diabetic nephropathy^509^)	Diabetic Nephropathy; Chronic Kidney Disease	Zemplar (paricalcitol) capsules; Placebo	PHASE2
	NCT01694160	COMPLETED (There were no statistically significant differences in T50 between the paricalcitol and placebo groups^510^)	Proteinuria	Paricalcitol	PHASE3
	NCT00208793	COMPLETED (Supplemental calcium and vitamin D3 increase TGFβ1 and TGFα expression in the normal-appearing colorectal mucosa of sporadic colorectal adenoma patient^511^)	Colorectal Adenomatous Polyps	DIETARY_SUPPLEMENT: Calcium and vitamin D3 combined; Placebo; Calcium; Vitamin D3	PHASE2
	NCT01371877	COMPLETED (Add-on therapy with high-dose vitamin D3 could be considered a safe and potentially beneficial immunomodulator in patients with chronic urticaria^512^)	Urticaria; Angioedema; Hives; Swelling	High Dose Vitamin D3; Low Dose Vitamin D3	NA
	NCT01974245	COMPLETED (Restoration of vitamin D status of patients undergoing dialysis promoted upregulation of CYP27B1 and VDR expression in monocytes and a decrease in circulating inflammatory markers^513^)	Chronic Kidney Disease	Cholecalciferol; Placebo	PHASE2
	NCT00986596	COMPLETED (No Results Posted)	Sarcopenia; Falls	DIETARY_SUPPLEMENT: vitamin D3; Placebo	NA
	NCT00470353	TERMINATED (No Reason Posted)	Colorectal Cancer	DIETARY_SUPPLEMENT:	NA
				Calcium carbonate; cholecalciferol;	
				immunohistochemical staining method; laboratory biomarker analysis; pharmacological study; biopsy	
	NCT05926570	COMPLETED (No Results Posted)	Drug Effect	Cinacalcet	PHASE4
	NCT03318029	COMPLETED (Moderate supplementation of cholecalciferol, in accordance with current recommendations, supports an adequate vitamin D status in adult women^514^)	Vitamin D Deficiency	DIETARY_SUPPLEMENT: Vitamin D supplementation; Placebo	NA
	NCT00258258	TERMINATED (No Reason Posted)	Multiple Myeloma and Plasma Cell Neoplasm	Paricalcitol; zoledronic acid	PHASE1
	NCT00217477	COMPLETED (No Results Posted)	Unspecified Adult Solid Tumor	Gemcitabine hydrochloride; paricalcitol	PHASE1
	NCT00634582	TERMINATED (No Reason Posted)	Metastatic Cancer; Prostate Cancer	Paricalcitol; immunoenzyme technique; laboratory biomarker analysis; PROCEDURE: Dual X-ray absorptometry; quality-of-life assessment	PHASE2
Pregnane X receptor (PXR)	NCT00621309	COMPLETED (No Results Posted)	Adverse Drug Interactions	Rifampicin; DIETARY_SUPPLEMENT: sulforaphane plus rifampicin; sulforaphane alone	PHASE1
	NCT02329405	COMPLETED (PXR activation elevates SBP)	Nonalcoholic Fatty Liver Disease	Rifampicin; Placebo	PHASE4
	NCT01690104	COMPLETED (PXR activation elevates SBP)	Blood Pressure Regulation	Rifampicin; Placebo	PHASE4
	NCT01293422	COMPLETED (PXR activation elevates SBP)	Glucose Metabolism	Rifampicin	PHASE4
	NCT00985270	COMPLETED (PXR activation elevates SBP)	Glucose Tolerance	Rifampicin; Placebo	PHASE4
	NCT05073627	COMPLETED (No Results Posted)	Healthy Volunteers	Dicloxacillin	PHASE1
	NCT05552196	COMPLETED (No Results Posted)	Healthy	Ponesimod; Carbamazepine	PHASE1
	NCT01550640	COMPLETED (At a dose of 1 μg/kg, remifentanil prior to induction of general anesthesia increases the risk of neonatal respiratory depression during first minutes after cesarean delivery but duration of clinical symptoms is short^515^)	Pregnancy; Cesarean Delivery; General Anesthesia	Remifentanil	NA
	NCT02629770	COMPLETED (No Results Posted)	Arthritis, Infectious; Bone Diseases, Infectious	Usual antibiotic treatment	NA
	NCT01548079	COMPLETED (Ursodeoxycholic acid treatment has ambivalent effects in NAFLD patients^516^)	Nonalcoholic Fatty Liver Disease; Morbid Obesity	Ursodeoxycholic Acid (UDCA)	NA
	NCT04840641	COMPLETED (No Results Posted)	Healthy Volunteers	Flucloxacillin	PHASE1
	NCT00258258	TERMINATED (No Reason Posted)	Multiple Myeloma and Plasma Cell Neoplasm	Paricalcitol; zoledronic acid	PHASE1
	NCT00217477	COMPLETED (No Results Posted)	Unspecified Adult Solid Tumor	Gemcitabine hydrochloride; paricalcitol	PHASE1
	NCT00634582	TERMINATED (No Reason Posted)	Metastatic Cancer; Prostate Cancer	Paricalcitol; immunoenzyme technique; laboratory biomarker analysis; dual X-ray absorptometry; quality-of-life assessment	PHASE2
Estrogen receptor-α (ERα)	NCT01874756	TERMINATED (No Reason Posted)	Schizophrenia	LY500307 150 mg; LY500307 75 mg; Placebo; LY500307 25 mg	PHASE2
Estrogen receptor-β (ERβ)	NCT02067741	TERMINATED (No Reason Posted)	Metastatic Breast Adenocarcinoma; Breast Cancer	CR1447	PHASE2
Glucocorticoid receptor (GR)	NCT00721201	COMPLETED (Mifepristone reduces insulin resistance in some individuals with adrenal incidentalomas and mild cortisol excess^517^)	Subclinical Cushing’s	Mifepristone	PHASE1; PHASE2
	NCT01990560	COMPLETED (No Results Posted)	Mild Hypercortisolism	Mifepristone	PHASE4
	NCT05062174	WITHDRAWN (Study results have not been submitted. This may be because the study is not done, the deadline for submitting results has not passed, this study is not required to submit results, or the sponsor or investigator has requested or received a certification to delay submitting the results)	BRCA1 Mutation; High-grade Serous Ovarian Cancer; TNBC - Triple- Negative Breast Cancer	Mifepristone 200 MG; Prophylactic mastectomy	NA
	NCT02989662	COMPLETED (No Results Posted)	Alcohol Use Disorder	Mifepristone; Placebo - Cap	PHASE1; PHASE2
	NCT03622112	COMPLETED (No Results Posted)	Asthma	AZD7594 DPI; AZD7594 DPI; AZD7594 DPI; AZD7594 DPI once daily; AZD7594 DPI; Placebo for AZD7594 once daily; FF 100 once daily (open-label)	PHASE2
	NCT01212588	TERMINATED (No Reason Posted)	Borderline Personality Disorder	Mifepristone; Placebo	PHASE2
	NCT03052400	TERMINATED (Administrative barriers and pandemic closures limited enrollment, curtailed several secondary outcome measures, and caused early termination of the study)	Type 2 Diabetes Mellitus; Insulin Resistance	Mifepristone 600 mg daily; Placebo	PHASE2
	NCT03697109	COMPLETED (Glucocorticoid Receptor Antagonism Upregulates Somatostatin Receptor Subtype 2 Expression in adrenocorticotropic hormone-Producing Neuroendocrine Tumors^518^)	Cushing Syndrome	Relacorilant; Placebo	PHASE3
	NCT04588688	TERMINATED (No Reason Posted)	Central Adrenal Insufficiency; Mifepristone	Mifepristone	PHASE2
	NCT00691067	COMPLETED (A moderate dose of mifepristone may have circumscribed cognitive-enhancing effects in chronic multisymptom illness^519^)	Chronic Multisymptom illness in Gulf War Veterans	Mifepristone; Placebos	PHASE4
	NCT03437941	COMPLETED (Enzalutamide combined with mifepristone was safe and well tolerated but did not meet its primary endpoint^520^)	Metastatic Castration-Resistant Prostate Cancer	CORT125281; Enzalutamide (Xtandi); Placebo	PHASE1; PHASE2
	NCT02762981	COMPLETED (GR modulation in patients with adrenocorticotropic hormone-secreting neuroendocrine tumors upregulates previously suppressed SSTR2s, resulting in tumor-specific antisecretory and anti-proliferative effects^518^)	Solid Tumors	Relacorilant with nab-paclitaxel	PHASE1; PHASE2
	NCT00285818	COMPLETED (No Results Posted)	Depression	Mifepristone; Placebo Oral Capsule	NA
	NCT02012296	COMPLETED (No Results Posted)	Hormone-resistant Prostate Cancer; Recurrent Prostate Cancer; Stage IV Prostate Cancer	Enzalutamide; mifepristone; laboratory biomarker analysis; pharmacological study	PHASE1; PHASE2
	NCT03928314	TERMINATED (No Reason Posted)	Solid Tumor	ORIC-101; Nab-paclitaxel Nab-paclitaxel, 100 mg/m^2; Relacorilant, 150 mg QD	PHASE1
	NCT02967159	COMPLETED (No Results Posted)	Chronic Obstructive Pulmonary Disease (COPD) Asthma	Abediterol; AZD7594; AZD7594/abediterol; AZD7594 and abediterol	PHASE1
	NCT03248713	TERMINATED (No Reason Posted)	Nicotine Dependence	Mifepristone; Placebo	EARLY_PHASE1
	NCT00522678	COMPLETED (No Results Posted)	Asthma	GW685698X; Placebo	PHASE1
Mineralocorticoid receptor (MR)	~558 studies, https://clinicaltrials.gov/search?term=%E2%80%9CMineralocorticoid%20receptor%E2%80%9D&intr=drug				
Progesterone receptor (PR)	~426 studies, https://clinicaltrials.gov/search?term=%22Progesterone%20receptor%22&intr=drug				
Androgen receptor (AR)	~365 studies, https://clinicaltrials.gov/search?term=%22Androgen%20receptor%22&intr=drug

**Table 2:** Summary of ongoing clinical trials of nuclear receptor-targeted drugs

Target	NCT number	Study satus	Conditions	Agents	Phases
Retinoic acid receptor-α (RARα)	NCT04793919	RECRUITING	Acute Promyelocytic Leukemia	Mylotarg; Arsenic Trioxide; All-trans retinoic acid (ATRA)	PHASE2
	NCT04113863	RECRUITING	Breast Neoplasm Female	ATRA; Anastrozole 1 mg	PHASE2
	NCT04460963	RECRUITING	Acute Myeloid Leukemia	Adrenomedullin	NA
	NCT02688140	ACTIVE_NOT_RECRUITING	Acute Promyelocytic Leukemia	Arsenic trioxide; Idarubicin; Cytarabine; Tretinoin; Mitoxantrone; Mercaptopurine; Methotrexate	PHASE3
Peroxisome proliferator-activated receptor-α (PPARα)	NCT05781698	RECRUITING	Inflammatory Bowel Diseases	Mesalamine; Fenofibrate	PHASE2
	NCT05753267	RECRUITING	Inflammatory Bowel Diseases	Mesalamine; Fenofibrate	PHASE2;
					PHASE3
	NCT06125587	ENROLLING_BY_INVITATION	Polycystic Ovary Syndrome	Chiglitazar; Metformin	PHASE2;
					PHASE3
	NCT06155331	RECRUITING	Breast Cancer Stage 2 and 3	Fenofibrate; Placebo; Doxorubicin; Cyclophosphamide	PHASE4
	NCT05542615	RECRUITING	Liver Cirrhosis; Hepatitis C, Chronic; Epigenetic Disorder	Prolonged-Release Pirfenidone	PHASE2
Peroxisome proliferator-activated receptor-γ (PPAR-γ)	NCT02391519	RECRUITING	Pregnancy; IUGR; Preeclampsia	NA	NA
	NCT06125587	ENROLLING_BY_INVITATION	Polycystic Ovary Syndrome	Chiglitazar; Metformin	PHASE2;
					PHASE3
	NCT06010992	RECRUITING	Diabetes Mellitus, Type 2	Nitazoxanide	PHASE2
	NCT05542615	RECRUITING	Liver Cirrhosis Hepatitis C, Chronic Epigenetic Disorder	Prolonged-Release Pirfenidone	PHASE2
	NCT03468556	NOT_YET_RECRUITING	NASH - Nonalcoholic Steatohepatitis	SNP-610; Placebo Oral Tablet	PHASE2
	NCT04114136	RECRUITING	Melanoma;NSCLC; Hepatocellular Carcinoma; Urothelial Cancer; Gastric Adenocarcinoma; HNSCC;Esophageal Adenocarcinoma; Microsatellite Instability-High Solid Malignant Tumor	Nivolumab or Pembrolizumab; Metformin; Rosiglitazone	PHASE2
	NCT05767671	RECRUITING	Acute Respiratory Distress Syndrome;Ventilator Associated Pneumonia	OTHER: Non-Bronchoscopic Mini Bronchoalveolar Lavage	NA
	NCT06054607	RECRUITING	Aerobic Endurance; Metabolism	DIETARY_SUPPLEMENT: High-amylose maize starch+ acetate/butyrate; Low-amylose maize starch	NA
RAR-related orphan receptor-α (RORα)	NCT04631341	NOT_YET_RECRUITING	Cardiovascular Diseases	DIETARY_SUPPLEMENT: Melatonin	NA
Steroid hormone receptor cnr14 (Sex-1)	NCT06123650	RECRUITING	Stroke, Acute; Dysphagia; Pneumonia	DEVICE: Repetitve transcranial magnetic stimulation; Sham transcranial magnetic stimulation	NA
	NCT06123650	RECRUITING	Stroke, Acute; Dysphagia; Pneumonia	DEVICE: Repetitve transcranial magnetic stimulation; Sham transcranial magnetic stimulation	NA
Farnesoid X receptor (FXR)	NCT05718349	RECRUITING	Cholestasis; Icterus; Cholangiocarcinoma; Pancreatic Neoplasms; Liver Metastasis Colon Cancer	OTHER: Biospecimen sampling	NA
	NCT03004118	ACTIVE_NOT_RECRUITING	Functional Dyspepsia	DIETARY_SUPPLEMENT: Nutridrink; DEVICE: High resolution manometry probe; DEVICE: perfusion catheter; Placebo Oral Tablet; Ursochol oral tablet; Duodenal fluid aspiration catheter; PROCEDURE: Duodenogastroscopy; Blood sample	PHASE4
	NCT04939051	RECRUITING	Barrett Esophagus	PROCEDURE: Biospecimen Collection; Esophageal Biopsy; Esophagogastroduodenoscopy; Liver Ultrasonographic Elastography; BIOLOGICAL: Obeticholic Acid; Placebo Administration; Questionnaire Administration	PHASE2
	NCT05740631	RECRUITING	Healthy	Ocaliva; Placebo	NA
	NCT05639543	RECRUITING	Alcohol Associated Hepatitis	INT-787; Placebo	PHASE2
Vitamin D receptor (VDR)	NCT05802433	RECRUITING	Vitamin D Deficiency	DIETARY_SUPPLEMENT: Vitamin D3 supplement	NA
	NCT05758259	ENROLLING_BY_INVITATION	Acne Vulgaris; Vitamin D	Combination oral and topical vitamin D; Oral placebo and topical cholecalciferol; Oral placebo and basic ingredient placebo topical vitamin D	PHASE4
	NCT03513770	RECRUITING	Venous Distension Reflex; Blood Pressure	OTHER: Wrist-to-elbow (W‒E) occlusion; ketorolac tromethamine; saline control	EARLY_PHASE1
	NCT02172651	RECRUITING	Colon Cancer (Stage I-III); Stage IV Colon Cancer With Resectable Liver Metastases	Vitamin D3; Placebo	EARLY_PHASE1
	NCT03467789	ACTIVE_NOT_RECRUITING	Basal Cell Carcinoma; Basal Cell Nevus Syndrome	Dietary Vitamin D3 pretreatment; RADIATION: Photodynamic therapy; Serum Maintenance Vitamin D3	PHASE1
	NCT06209268	NOT_YET_RECRUITING	Sepsis	Paricalcitol injection; Placebo	NA
	NCT05043116	RECRUITING	Asthma in Children	DIETARY_SUPPLEMENT: Cholecalciferol D3; Oral placebo suspension	PHASE2
	NCT01787409	RECRUITING	Aggressive Non-Hodgkin Lymphoma; Anaplastic Large Cell Lymphoma; Angioimmunoblastic T-Cell Lymphoma; Chronic Lymphocytic Leukemia; Diffuse Large B-Cell Lymphoma; Enteropathy-Associated T-Cell Lymphoma; B-Cell Lymphoma; Nasal Type Extranodal NK/T-Cell Lymphoma; Mediastinal (Thymic) Large Hepatosplenic T-Cell Lymphoma; Mature T-Cell and NK-Cell Non-Hodgkin Lymphoma; Peripheral T-Cell Lymphoma, Not Otherwise, Specified; Primary Cutaneous Anaplastic Large Cell Lymphoma;Refractory Anaplastic Large Cell Lymphoma; Small Lymphocytic Lymphoma; Subcutaneous Panniculitis-Like T-Cell Lymphoma	DIETARY_SUPPLEMENT: Cholecalciferol	NA
	NCT03004118	ACTIVE_NOT_RECRUITING	Functional Dyspepsia	DIETARY_SUPPLEMENT: Nutridrink; perfusion catheter; Placebo Oral Tablet; Ursochol oral tablet; Duodenal fluid aspiration catheter; PROCEDURE: Duodenogastroscopy; Blood sample	PHASE4
	NCT04677816	RECRUITING	Triple Negative Breast Cancer; Vitamin D Deficiency; Invasive Breast Cancer	Standard of Care Neoadjuvant Chemotherapy (NAC); DIETARY_SUPPLEMENT: Vitamin D3; Drug Diary	PHASE2
	NCT06279910	RECRUITING	SARS-CoV 2 Pneumonia	Calcifediol	NA
	NCT03520790	ACTIVE_NOT_RECRUITING	Pancreatic Cancer	Gemcitabine; Nab-paclitaxel; Paricalcitol; Placebo	PHASE1; PHASE2
	NCT05981534	RECRUITING	Vitamin D Deficiency; Sarcopenia; Knee Osteoarthritis	Vitamin D; Placebo	NA
	NCT05720273	RECRUITING	Chronic Kidney Disease-Mineral and Bone Disorder	Palicalcitol	PHASE4
	NCT06289257	NOT_YET_RECRUITING	Endometriosis	DIAGNOSTIC_TEST: Vitamin D level; Vitamin D receptor	NA
	NCT06327581	RECRUITING	Alopecia Areata	Lactic Acid; vit D; Triamcinolone Acetonide; Saline	NA
	NCT05839938	ENROLLING_BY_INVITATION	Allergic Conjunctivitis	Vitamin D; Placebo	NA
	NCT05497596	RECRUITING	Urticaria	Vitamin D; Placebo	NA
	NCT05633472	RECRUITING	Postacute COVID-19 Syndromes	Vitamin D; Placebo	NA
	NCT05523986	RECRUITING	Atopic Dermatitis	Vitamin D; Placebo	NA
	NCT03613116	ACTIVE_NOT_RECRUITING	Vitamin D Deficiency; Cognitive Decline	DIETARY_SUPPLEMENT: Vitamin D3	PHASE2
	NCT02876211	RECRUITING	Anemia	Paricalcitol; Epoetin beta; Placebo	PHASE4
Pregnane X receptor (PXR)	NCT03004118	ACTIVE_NOT_RECRUITING	Functional Dyspepsia	DIETARY_SUPPLEMENT: Nutridrink; DEVICE: High resolution manometry probe; DEVICE: perfusion catheter; Placebo Oral Tablet; Ursochol oral tablet; DEVICE: Duodenal fluid aspiration catheter; PROCEDURE: Duodenogastroscopy; Blood sample	PHASE4
Hepatocyte nuclear factor-4-α (HNF4α)	NCT06092112	NOT_YET_RECRUITING	Advanced Hepatocellular Carcinoma	CD-801	EARLY_PHASE1
	NCT06418659	NOT_YET_RECRUITING	Advanced Hepatocellular Carcinoma	CD-801	EARLY_PHASE1
Estrogen receptor-α (ERα)	NCT02694809	RECRUITING	Ductal Breast Carcinoma In Situ; Postmenopausal	Conjugated Estrogens/Bazedoxifene; Laboratory Biomarker Analysis; Pharmacological Study; Placebo; Quality-of-Life Assessment; Questionnaire Administration	PHASE2
	NCT04174352	RECRUITING	ERα+ Breast Cancer; ESR1 Gene Mutation	Tamoxifen; DIAGNOSTIC_TEST: FES PET/CT	EARLY_PHASE1
Glucocorticoid receptor (GR)	NCT03674814	ACTIVE_NOT_RECRUITING	Prostate Cancer	Enzalutamide; Relacorilant	PHASE1
	NCT02788981	ACTIVE_NOT_RECRUITING	Breast Cancer	Mifepristone; Placebo; Nab-Paclitaxel	PHASE2
	NCT04452500	RECRUITING	PTSD	CORT108297; Placebo	PHASE2
	NCT05726292	RECRUITING	Prostate Cancer; Prostate Adenocarcinoma	Relacorilant; Enzalutamide; Placebo (Sugar Pill); Androgen Deprivation Therapy; Radical Prostatectomy	PHASE2
	NCT04601038	RECRUITING	Mild Cognitive Impairment; Alzheimer Disease; Memory Impairment	CORT108297; Placebo	PHASE2
	NCT04308590	ACTIVE_NOT_RECRUITING	Hypercortisolism	Relacorilant; Placebo	PHASE3
Mineralocorticoid receptor (MR)	~558 studies, https://clinicaltrials.gov/search?term=%E2%80%9CMineralocorticoid%20receptor%E2%80%9D&intr=drug				
Progesterone receptor (PR)	~426 studies, https://clinicaltrials.gov/search?term=%22Progesterone%20receptor%22&intr=drug				
Androgen receptor (AR)	~365 studies, https://clinicaltrials.gov/search?term=%22Androgen%20receptor%22&intr=drug				

### Targeting nuclear receptors in cancer

NRs, especially those that bind to three steroid hormones, namely, estrogen, progesterone and androgen, play a significant role in the development of human malignancies, particularly breast cancer,^355,356^ ovarian cancer^357^ and prostate cancer.^358^ In recent years, NR-targeted drugs have attracted widespread attention as novel therapeutic strategies^359–361^ that play a significant role in inhibiting the growth and progression of many types of cancer.

Within the nuclear receptor superfamily, ERs were the first nuclear receptors to be described. Breast cancer is the most common cancer in women, with ER^+^ breast cancer accounting for approximately 75% of all cases. Numerous studies have confirmed that ERα plays a causative role in carcinogenesis. As a result, an increasing number of ERα-based targeted medicines have entered preclinical and clinical trials (Table 1), and some have even received FDA approval. This includes directly antagonizing the estrogen receptor via SERMs and selective estrogen receptor degraders (SERDs). These drugs include ER antagonists, selective ER modulators and aromatase inhibitors.^362^ ER antagonists competitively inhibit the ER, whereas selective ER modulators function as partial agonists or antagonists on the basis of their tissue affinity. Aromatase inhibitors decrease estrogen production by blocking aromatase in tissues such as the ovaries and adipose tissue.^363,364^ Tamoxifen is the most widely used antiestrogen for hormone-dependent breast cancer and is indicated for various treatment settings. Evidence shows that patients with estrogen receptor-positive tumors are more likely to benefit from tamoxifen. FDA-approved treatments include treating breast cancer in both females and males, providing adjuvant therapy after surgery and radiation, treating female patients with ductal carcinoma in situ (DCIS) postsurgery and radiation to lower the risk of invasive breast cancer and reduce the risk of breast cancer in certain high-risk patients.^365,366^ In addition to tamoxifen, many other SERMs, including raloxifene, lasofoxifene, bazedoxifene and fulvestrant, have been developed and used for breast cancer treatment.^363^ These drugs have also been used to treat other tumors with high estrogen expression. In addition to hormone-dependent breast cancer, antiestrogen medications such as tamoxifen and aromatase inhibitors have shown efficacy in managing conditions such as endometrial cancer and certain types of ovarian cancer.^367,368^ Although estrogen levels are low in prostate tissue, ER expression has been detected in some prostate cancer patients.^369^ ER activation is believed to be associated with the proliferation and metastasis of prostate cancer cells.^370^ Thus, antiestrogen therapy targeting the ER has also been proposed as a new treatment strategy for prostate cancer. For example, in males with advanced prostate cancer, an ERα agonist, GTx-758, has been shown to lower testosterone with fewer side effects associated with androgen-deprivation therapy.^371^

Bidedoxifene is the most recent generation of SERMs. Fanning and colleagues demonstrated that bazedoxifene works in concert with palbociclib, a CDK4/6b inhibitor, and is more effective than tamoxifen against specific ERα mutants, such as Y537S and D538G. A phase II clinical trial in patients with ductal carcinoma in situ [NCT02694809] and a phase I/II clinical trial on bazedoxifene in combination with palbociclib in hormone receptor-positive breast cancer [NCT02448771] are two examples of the numerous clinical trials that have examined bazedoxifene across a variety of cancer types. Degradation of this receptor by SERDs is another tactic to target ERs. Fulvestrant is the most researched SERD, and its clinical application has been growing. Clinical trials have combined fulvestrant with CDK4/6 inhibitors, such as ribociclib in men and postmenopausal women with advanced breast cancer [NCT02422615] and palbociclib in hormone receptor+HER2-metastasized breast cancer after endocrine failure [NCT01942135], as well as phosphatidylinositol 3-kinasitol kinase (PI3K)/AKT/mTOR pathway inhibitors, such as pictilisib, in advanced or metastatic breast cancer in participants resistant to aromatase inhibitor therapy [NCT01437566].

The abnormal expression of AR in prostate cancer cells makes anti-AR drugs a logical treatment strategy.^372^ These drugs inhibit AR function through various mechanisms, reducing androgen stimulation of tumor cells. Among the most common anti-AR agents are AR antagonists such as abiraterone and enzalutamide. These drugs competitively bind to the AR, preventing androgen attachment and inhibiting receptor activity. Abiraterone has been shown to prolong survival in patients with metastatic castration-resistant prostate cancer (mCRPC).^373^ In the context of metastatic castration-sensitive prostate cancer (mCSPC), a combination treatment involving abiraterone, prednisone and docetaxel has favorable effects.^374^ Furthermore, for metastasis-free survival, combining enzalutamide with leuprolide has shown superior outcomes compared with leuprolide alone in high-risk patients with biochemical recurrence. Additionally, enzalutamide monotherapy has outperformed leuprolide monotherapy.^36^ The prostate cancer community has recently focused much attention on a number of newly created AR proteolysis-targeting chimeras (PROTACs), which were created as SARDs. ARV-110 [NCT03888612], the first AR PROTAC to reach clinical trials, is now being used in patients with metastatic CRPC and is undergoing a phase I/II clinical trial. In contrast to clinically licensed AR antagonists, ARD-61, a novel AR PROTAC, has been shown to efficiently promote on-target AR degradation, elicit more potent antiproliferative and proapoptotic effects, and reduce downstream AR target gene expression in prostate cancer cells. The most effective AR PROTACs may eventually be utilized extensively in cancer patients, and we expect more advancements to the clinical trial stage.

Additionally, RARs and their heterodimer partners, such as PPARs and RXR, which are involved in vitamin A metabolism, cell differentiation and immune regulation, also have therapeutic potential.^375,376^ Aberrant RXR signaling can promote cancer development by disrupting normal cellular homeostasis and enhancing oncogenic processes. RXR agonists have shown potential in treating various advanced cancers in clinical trials. IRX4204, in combination with erlotinib, is under investigation for previously treated advanced non-small cell lung cancer (NSCLC) [NCT02991651]. NRX194204 is being studied for its efficacy in castration- and taxane-resistant prostate cancer [NCT01540071] and advanced NSCLC [NCT00964132]. Additionally, bexarotene is being evaluated for the treatment of metastatic breast cancer and relapsed or refractory cutaneous T-cell lymphoma in patients with stage III or IV disease [NCT00003752, NCT00514293, NCT01134341, NCT00316030]. These studies underscore the therapeutic potential of RXR agonists in managing resistant and advanced malignancies. PPAR agonists have shown promising antitumor effects across a range of cancers, either as standalone treatments or in combination therapies. Troglitazone has shown efficacy in prostate cancer and liposarcoma [NCT00003058], whereas pioglitazone is being studied for advanced solid tumors [NCT02133625], pancreatic cancer [NCT01838317], and thyroid cancer [NCT01655719], often with chemotherapy. Inolitazone combined with paclitaxel is under investigation for advanced anaplastic thyroid cancer [NCT02152137]. Rosiglitazone is being explored for the treatment of liposarcoma [NCT00004180], metastatic thyroid cancer [NCT00098852], and androgen-dependent prostate cancer [NCT00182052]. These trials demonstrate the therapeutic potential of PPAR agonists in treating challenging malignancies. Retinoic acid, a natural RAR agonist, regulates the expression of genes related to cell growth. Studies suggest that RAR/RXR agonists may have antitumor effects on prostate cancer. For example, the RAR agonist adapalene has been shown to induce a tumor-suppressive senescence-associated secretory phenotype (SASP) in prostate cancer cells. In combination with docetaxel, adapalene enhanced tumor suppression and improved natural killer (NK) cell-mediated clearance in preclinical models. This approach also increased the efficacy of NK cell infusion in mice with human prostate cancer, leading to greater tumor suppression.^377^

REV-ERBs, including REV-ERBα (NR1D1) and REV-ERBβ (NR1D2), are key regulators of the circadian clock. Notably, many tumor types exhibit elevated expression of REV-ERBβ, regardless of ERBB2 or ER status, whereas nonmalignant mammary epithelial cells primarily express REV-ERBα. High levels of REV-ERBβ in cancer cells have been linked to reduced sensitivity to chloroquine via the modulation of autophagy, suggesting a role in therapy resistance. REV-ERB agonists, such as SR9009 and SR9011, selectively target cancer cells and oncogene-induced senescent cells, such as melanocytic naevi, without impacting normal cell viability. These agonists have shown promising anticancer effects, including reduced glioblastoma growth and improved survival in mice, without causing significant toxicity.^378^

### Targeting nuclear receptors in metabolic-related diseases

Metabolic diseases such as nonalcoholic fatty liver disease (NAFLD), obesity, type 2 diabetes and atherosclerosis represent a growing global health burden.^379,380^ These conditions are frequently associated with dysregulated lipid or glucose metabolism.^381,382^ NRs play crucial roles in maintaining metabolic homeostasis by controlling the expression of genes involved in lipid, glucose and energy metabolism. Among NRs, PPARs, farnesoid X receptor (FXR), liver X receptor (LXR) and pregnane X receptor (PXR) have been identified as promising therapeutic targets. Drugs that modulate these receptors, such as PPAR agonists and FXR agonists, have demonstrated promising activity in clinical trials for the treatment of metabolic disorders, particularly NAFLD. Here, we explore current and emerging therapies that target these receptors.

One of the most well-known nuclear receptors is the PPAR family. PPAR agonists, such as pioglitazone and rosiglitazone, can increase insulin sensitivity in individuals with T2D. By activating PPARγ, these drugs enhance glucose uptake and lipid storage, which helps to lower blood sugar levels and improve metabolic control. However, these drugs are associated with certain risks, such as weight gain and fluid retention, necessitating careful patient management. PPARα agonists, such as fibrates, have been used clinically for 35 years to regulate blood lipids and prevent atherosclerosis.^383^ Pemafibrate was introduced in Japan in June 2018 and has since been demonstrated to be effective in inhibiting VLDL secretion and enhancing triglyceride (TG) clearance by activating LPL.^384^ In mice fed a high-fat diet, it suppressed postprandial hyperlipidemia by downregulating the mRNA expression of the intestinal cholesterol transporter NPC1L1 and upregulating genes involved in β-oxidation to inhibit VLDL secretion from the liver. In a 4-week trial with dyslipidemic patients (0.4 mg/day), pemafibrate significantly reduced fasting and nonfasting serum TG, RemL-C, and apo B-48 levels, with a marked reduction in the postprandial TG AUC, indicating improved postprandial hypertriglyceridemia.^385^ Similar results were observed in diabetic patients treated with pemafibrate.^386^ PPARγ and PPARδ regulate energy utilization and insulin sensitivity in skeletal muscle and adipose tissue. High insulin levels accelerate the development of NAFLD through a PPARγ-dependent pathway.^387^ PPARγ agonists, thiazolidinediones, have been used as insulin sensitizers in diabetic patients.^388^ PPARδ agonists improved the pathological manifestations of nonalcoholic steatohepatitis (NASH) without affecting body weight. The dual PPARα/δ agonist elafibranor (GFT505) improved glycemic and lipid metabolism in NASH patients in a phase II clinical trial, reduced liver inflammation and did not exacerbate fibrosis, demonstrating good tolerability. Phase III clinical trials targeting NASH patients [NCT02704403] are ongoing. A phase II clinical trial of the dual PPARα/γ agonist Saroglitazar [NCT03061721] in NASH patients was completed in April 2020. Midterm reports revealed not only regression of NASH tissue lesions but also decreased serum ALT levels, indicating improved liver function. The Indian pharmaceutical company Zydus Cadila has submitted a marketing application for this drug.

Another good NR drug target is FXR, which regulates bile acid metabolism and lipid homeostasis. FXR activation improves lipid metabolism and reduces liver inflammation, offering a new avenue for managing metabolic liver disorders.^389–391^ Obeticholic acid, an FXR agonist also known as INT-747, is approved for treating NAFLD and NASH^392^ [NCT02548351]. Owing to its ability to lower ALP levels and combat cholestasis and fibrosis, obeticholic acid (Ocaliva) was initially approved as a treatment for primary biliary cholangitis. In a phase III clinical trial for NASH [NCT02548351], obeticholic acid improved liver function and reduced tissue pathology, although it exhibited some side effects, such as pruritus and increased low-density lipoprotein levels. Given that pruritus can be managed symptomatically and that dyslipidemia can be addressed by the concomitant use of statins, the United States Food and Drug Administration has approved obeticholic acid as the primary treatment for NASH. Several other FXR agonists with diverse physicochemical characteristics, including bile acid derivatives, nonbile acid-derived steroidal FXR agonists, nonsteroidal FXR agonists and partial FXR agonists, are also progressing through advanced stages of clinical development. The noncarboxylic acid steroidal FXR agonist EDP-305 has been shown to modulate many pathways that trigger antilipogenic, anti-inflammatory and antifibrotic gene signatures. This resulted in hepatoprotective and antisteatotic effects in a mouse model of NASH.^393^ Patients with intestinal failure require more complicated care when they have parenteral nutrition (PN)-associated cholestasis (PNAC). By restoring hepatic FXR signaling, GW4064 protected animals against PNAC by increasing the expression of sterol and phospholipid transporters and canalicular bile and suppressing the recruitment and activation of macrophages. These findings suggest that increasing FXR activity could be used as a therapeutic approach to treat or prevent PNAC.^394^

In addition to PPAR and FXR, LXR is involved in cholesterol metabolism and can be targeted for atherosclerosis treatment. LXR agonists have been shown to increase cholesterol efflux and reduce the development of atherosclerotic plaques in preclinical models.^395^ However, treating patients with medications that increase cholesterol is not a practical option for metabolic illnesses; instead, methods for separating the advantageous effects of LXR ligands from unfavorable side effects must be investigated. Taken together, these examples highlight the growing potential of NR-targeted drugs in addressing the underlying causes of metabolic disorders, offering more precise and effective treatments than traditional approaches do.

### Targeting nuclear receptors in cardiovascular diseases

The targeting of NRs for the treatment of cardiovascular diseases has gained significant attention because of the pivotal role these receptors play in regulating the expression of genes involved in metabolic, inflammatory and lipid processes.^361,396–398^ Nuclear receptors, such as PPARs, LXR and FXR, are key regulators of lipid metabolism, glucose homeostasis and inflammation, all of which are critical factors in the development of cardiovascular diseases. By modulating these receptors, it is possible to control pathological processes such as atherosclerosis, hypertension and other cardiovascular disorders.

The NRs RORα and REV-ERBα are key components of the circadian system and regulate biological rhythms, glucose metabolism, lipid metabolism, vascular inflammation and fibrinolysis.^399^ RORα is instrumental in endothelial progenitor cell (EPC) differentiation and promotes angiogenesis, tissue repair and vascular stability by inducing erythropoietin production and activating AMPK.^400^ RORα mediates the protective effects of melatonin, including its anti-inflammatory, antioxidant and antiapoptotic effects, making it a potential therapeutic target for atherosclerosis and endothelial dysfunction.^401,402^ In hypertensive rats, melatonin altered clock gene expression patterns, maintaining high levels of REV-ERBα mRNA, which regulated blood pressure rhythms. This effect is linked to melatonin receptor activity and may involve posttranslational mechanisms.^403^ Increased RORα expression, which is stimulated by melatonin and the sympathetic nervous system, also protects against programmed hypertension. A number of small-molecule agonists for RORα and REV-ERBα, such as the pharmacological agents SR9009 and GSK4112 for REV-ERBα, have been developed^404^ and are available for use in cellular and animal experiments, but prospective studies on the impact of stable circadian rhythms on CHD and heart failure are still limited.^405,406^ When considered collectively, the bulk of current research points to considerable clinical value and therapeutic potential for medications that target RORα and REV-ERBα to treat hypertension and atherosclerosis.

Several studies have highlighted the function of RXRs in cardiovascular disease. Treatment with 9cRA or the synthetic RXR agonist LGD1069 (bexarotene) has been shown to reduce oxidative stress-induced cell damage and preserve the mitochondrial membrane potential.^407^ In heart failure, oxidative stress activates angiotensin-II (AngII), leading to harmful cardiac remodeling and dysfunction. Bexarotene treatment of cultured rat aortic smooth muscle cells inhibited AngII-driven inflammation and p38 MAPK activation, suggesting that RXR activation has protective effects on cardiac disease.^408^ ERRα and ERRγ are essential for normal postnatal cardiovascular function, whereas ERRβ supports maximal ATP generation in contracting cardiomyocytes. ERR agonists help maintain oxidative metabolism, which protects the heart against pressure overload-induced heart failure (HF) in vivo. Using a structure-based design, Xu and colleagues synthesized two pan-ERR agonists, SLU-PP-332 and SLU-PP-915, each of which significantly improved the ejection fraction, reduced fibrosis and enhanced survival in models of pressure overload-induced HF without affecting cardiac hypertrophy.^409^ Targeting other nuclear receptors, such as RARs, PPARs, and LXRs, has also shown promise for ameliorating cardiovascular disease.^410,411^ These findings provide strong pharmacological evidence supporting the potential of targeting NRs for cardiovascular disease management.

### Targeting nuclear receptors in central nervous system disorders

Alzheimer’s disease (AD), PD, Huntington’s disease (HD), multiple sclerosis (MS), cerebral ischemia, stroke and other conditions are common central nervous system disorders that currently affect approximately one hundred million people worldwide. Compelling evidence suggests that nuclear receptors are important in these disorders and that targeting these receptors has significant therapeutic potential.^353^

Liver X receptor LXRs are key regulators of cholesterol trafficking in the central nervous system (CNS), working with ApoE to facilitate cholesterol recycling and efflux, which are essential for maintaining CNS homeostasis. Dysregulation of LXR signaling is linked to neurodegenerative diseases such as AD, PD and Niemann‒Pick type C disease. LXR activation offers therapeutic potential by modulating neuroinflammation in CNS disorders. In AD models, LXR agonists such as T0901317 reduce inflammation associated with amyloid-beta (Aβ) deposition and tau tangles by suppressing microglial activation and the expression of inflammatory markers such as iNOS, COX-2, and NF-κB. Similar effects are observed in PD models, where T0901317 decreases iNOS and COX-2 levels. In Niemann–Pick type C disease, LXR activation promotes cholesterol clearance, reduces cerebellar inflammation, and extends lifespan. LXRs also enhance blood‒brain barrier (BBB) integrity by increasing the expression of ABC transporters such as ABCB1, which prevents toxin entry into the brain. Natural LXR agonists (e.g., oxysterols) and RXR activation (e.g., with bexarotene) improve BBB function in coculture models, whereas LXR agonists support tissue regeneration in ischemic stroke by stabilizing the BBB and reducing inflammatory cytokines. Although preclinical results are promising, the clinical translation of LXR agonists is hindered by side effects, including elevated triglycerides and LDL cholesterol.^284^ Efforts to mitigate these issues include the development of selective LXRβ agonists, regional drug delivery, and the targeting of specific downstream pathways, making LXRs promising therapeutic targets for CNS diseases.

PPARs, particularly PPARγ, play pivotal roles in neuroprotection by regulating neuroinflammation, mitochondrial function, and antioxidant defenses. It is expressed in various CNS cell types, including neurons, astrocytes, oligodendrocytes, and microglia. Dysregulation of PPARs is associated with neurodegenerative diseases characterized by inflammation, such as AD and amyotrophic lateral sclerosis (ALS). PPARγ agonists, including endogenous ligands such as OA and synthetic compounds such as rosiglitazone and pioglitazone, have shown promise in preclinical models because they reduce inflammatory cytokines, increase IL-10 levels, and enhance mitochondrial function in conditions such as stroke, ischemia, and traumatic brain injury. Moreover, PPARγ activation has exhibited potential benefits in neuropsychiatric disorders (e.g., depression, anxiety, and mood disorders) and neurodegenerative diseases, with ongoing clinical trials exploring its applications in AD, PD, MS, and bipolar disorder^412^ [NCT00982202, NCT00736996, NCT00265148, NCT00733785, NCT00688207, NCT01280123]. However, mixed clinical outcomes highlight gaps in understanding the mechanisms of PPARγ within the CNS.^413^ Further research aimed at optimizing PPARγ activity may unlock its full therapeutic potential for treating neurodegenerative and neuropsychiatric conditions.

The GR also plays a critical role in neuroinflammation, neuroprotection, and brain homeostasis. Its anti-inflammatory effects in the brain are mediated through interactions with AP-1 and NF-κB, limiting their transcriptional activity.^414^ GR also regulates brain-derived neurotrophic factor (BDNF), promotes neurogenesis, neuronal survival, and synaptic plasticity, and contributes to memory, aging, and behavior. Additionally, GR supports BBB integrity by upregulating tight junction proteins and establishing metabolic barriers to xenobiotics.^415,416^ GR dysfunction has been implicated in various neuropathologies, including AD, HD and epilepsy. Abnormal GR expression in AD is correlated with hippocampal overexpression, impaired cortisol feedback, and memory deficits.^417^ Similarly, GR overexpression in epilepsy has been associated with reactive gliosis and coregulation with PXR in epileptic brain endothelial cells. GR dysregulation also affects HD-related changes, such as increased cortisol secretion and muscular atrophy, alongside reduced GR expression in the hypothalamic and pituitary regions. GR abnormalities also underlie mood disorders such as depression and anxiety, as dysfunction in GR and MR signaling leads to maladaptive stress responses, dendritic retraction, and impaired serotonin activity.^418,419^ GR dysregulation in the prefrontal cortex and hippocampus has been linked to anxiety and depression-like behaviors in animal models. Pharmacological modulation of the GR provides therapeutic opportunities. GR agonists, such as dexamethasone and methylprednisolone, are widely used to treat neurological conditions such as gliomas, MS, and traumatic brain injuries. Dexamethasone reduces edema, prevents cerebrovascular leakage, and upregulates tight junction proteins at the BBB.^420,421^ Methylprednisolone improves memory and long-term potentiation at low doses but has dose-dependent effects, with high doses alleviating motor deficits in MS models but not cognition.^422,423^ GR antagonists, such as mifepristone and relacorilant, have shown efficacy in treating mood disorders, neurodegenerative conditions, and PTSD. Mifepristone restores serotonin signaling and alleviates depression-like behaviors, whereas relacorilant is undergoing phase II and III trials for conditions such as Cushing syndrome and prostate cancer^424,425^ [NCT036748142019, NCT036971092019]. These findings underscore the potential of targeting GRs in a range of CNS disorders.

Estrogens and their receptors play critical roles in neuroprotection and the regulation of inflammatory and neurodegenerative processes in the CNS. Owing to their antiapoptotic, antioxidative, and anti-inflammatory properties, estrogens provide early protection against ischemic injury. ERα and ERβ agonists, such as diarylpropionitrile (DPN), have demonstrated neuroprotective effects by mitigating damage in ischemic models, including the caudate nucleus and CA1 region of ovariectomized mice and the striatum of male mice. The membrane receptor GPER-1 also contributes to ischemic protection, with its agonist G1 offering estrogen-like neuroprotection, particularly in males, as suggested by sex-dependent differences in GPER-1 expression. In addition to estrogens, NRs such as Nr4a2 and bile acid receptors play vital roles in alleviating neuroinflammation and neuropathic pain. Nr4a2 helps regulate depressive-like behaviors by preventing structural and functional damage to anterior cingulate cortex (ACC) microglia and excitatory neurons during chronic neuroinflammation.^426^ Its upregulation through the dopaminergic agonist pramipexole has demonstrated neuroprotective effects on dopaminergic neurons in Parkinson’s disease.^427^ Similarly, bile acids activate TGR5 and FXR, alleviating mechanical hyperalgesia by reducing glial activation and neuronal sensitization in the spinal dorsal horn. The presence of bile acid synthesis enzymes in spinal astrocytes suggests an endogenous mechanism for decreasing nociceptive neuron transmission and mechanical hypersensitivity, emphasizing bile acid receptors as promising targets for neuropathic pain relief.^428^ In MS, Th17-driven inflammation is aggravated by RORγt overexpression, which promotes Th17 cell differentiation. By suppressing RORγt, vitamin A has shown therapeutic potential for MS^429^[NCT01225289]. Collectively, these findings highlight the diverse and pivotal roles of estrogens and nuclear receptors in neuroprotection, inflammation regulation, and the development of innovative therapeutic strategies.

### Targeting nuclear receptors in infectious diseases

In recent decades, global spending on infectious diseases has increased significantly, with treatment costs rising faster than those for other diseases. Infectious diseases caused by bacteria, viruses, fungi, and parasites include common conditions such as pneumonia, influenza, gastroenteritis, hepatitis, malaria, and dengue fever, as well as diseases such as HIV/AIDS, tuberculosis, and COVID-19. NRs play a key role in regulating immune responses by modulating genes involved in inflammation, pathogen defense, and immune cell function, thus influencing the outcome of both bacterial and viral infections. Given the increasingly appreciated roles of NRs in the context of infections, targeting NRs may provide a new, largely unexplored area for infectious disease treatment.^430^

The role of FXR extends to hepatitis C virus (HCV) infection, where it, along with other NRs, such as HNF4α, PPARα, and PXR, reprograms hepatocyte metabolism, influencing glycolysis, ketogenesis, and drug metabolism.^431^ The targeting of FXR or PPARα in HCV infection has shown complex antiviral effects, with receptor inhibition reducing viral replication by reversing HCV-induced metabolic shifts. Furthermore, FXR agonists such as GW4064 have demonstrated potential in alleviating inflammation and preserving intestinal barrier function in experimental colitis, primarily through the modulation of NF-κB signaling.^432,433^ In addition, GW4064 has been reported to downregulate scavenger receptors via FXR and indirectly inhibits HCV entry into cells.^434^

In addition to their roles in viral infections, NRs are essential in bacterial infection. For example, PPARγ signaling is crucial for resolving *Staphylococcus aureus* (MRSA) infections and improving outcomes in sepsis models by modulating inflammation.^435^ PPARγ agonists such as pioglitazone have been shown to restore mitochondrial function and reduce oxidative stress in macrophages, highlighting their potential in treating inflammatory and infectious diseases.^436^ LXRs are involved in macrophage phagocytic responses, particularly during Salmonella Typhimurium infections.^437^ In a mouse model, LXR agonist treatment ameliorated clinical signs of Salmonella infection in a CD38-dependent manner.^438^ Recent transcriptomic studies investigating susceptibility to dengue virus in a Cuban cohort of African ancestry revealed increased gene expression of RXR in individuals who were more resistant to infection and suggested a protective role for LXR/RXR against dengue virus.^439^ In the case of tuberculosis (TB), NRs such as Nur77 and Nor1 are upregulated in response to *Mycobacterium tuberculosis* infection. Interestingly, the first-line TB drug rifampicin activates PXR, which can impair its effectiveness in infected macrophages, underscoring the complex interactions between NRs and drug efficacy.^440,441^ Rifampicin, a first-line anti-tuberculosis (TB) drug, is a potent activator of PXRs, which in turn compromises the effect of rifampicin in *M. tuberculosis*-infected human macrophages.^442^

NRs also influence fungal infections, with PPARγ playing a central role in defending against *Candida albicans* in the gastrointestinal tract. PPARγ ligands reduce fungal colonization through a dectin-1-dependent mechanism. Sepsis models have revealed a critical role for PPARγ in controlling inflammation and improving overall disease outcomes. PPARγ gene expression was downregulated in freshly isolated peripheral blood mononuclear cells (PBMCs) from human sepsis patients as well as in the lungs in a murine sepsis model.^443^ Treatment with PPARγ agonists improved sepsis outcomes, decreased proinflammatory cytokine levels, increased IL-10 levels, and improved mouse survival.^444^ Other PPAR isoforms have also been linked to decreased severity of sepsis through the downregulation of inflammation. In a cecal ligation and puncture rat sepsis model, treatment with a PPARβ/δ agonist inhibited signal transducer and activator of transcription 3 (STAT3) activation in AMs, reduced the inflammatory response, and prolonged rat survival.^445,446^

Parasitic worms are among the global health threats to billions of humans worldwide.^447,448^ Although several anthelmintic drugs, including benzimidazole, levamisole/morantel and ivermectin, have been used to treat these parasites, resistance frequently occurs. Therefore, identifying novel therapeutic targets in parasitic diseases is urgently needed. Nuclear receptors, which are well-known therapeutic targets in humans, are currently being investigated in parasitic worms because of their widespread distribution and important functions in controlling transcriptional networks involved in metabolism and development.^449^ Among them, DAF-12 is a nuclear receptor that is necessary for the typical development of nematodes, including the crucial infectious stage. In free-living nematodes such as *C. elegans*, a lack of DAF-12 ligands (such as dafachronic acid (DA)) under stress conditions promotes entry into the L3d (dauer diapause) state, whereas supplementation with DAF-12 ligands in prosperous environments leads to their exit from L3d and the beginning of reproductive development.^450^ In contrast, in parasitic nematodes such as S. stercoralis, the absence of a DAF-12 ligand leads to an L3d ortholog stage, termed the infectious L3 (iL3) stage, during preinfection, whereas the presence of a DAF-12 ligand in the definitive host can resume reproductive development.^451^

The mechanism underlying DAF-12-mediated cell fate decisions is similar to that of other nuclear receptors. Briefly, under favorable conditions or in a host-specific environment, DAF-12 binds with its ligand and is thus activated, which induces the transcription of target genes that facilitate metabolism and development. In contrast, under stress conditions, DAF-12 is inactive without ligand binding, resulting in transcriptional repression and subsequent development arrest and infection. Thus, strategies targeting DAF-12 or its ligands have potential for the treatment of parasitic diseases. In addition to exogenous DA, endogenous Δ7-DA was recently reported as a novel ligand of DAF-12 in S. stercoralis. The abundance of Δ7-DA is regulated by several enzymes, including DAF-36, DHS-16, and DAF-9, which govern the on/off switch of the reproduction, infection and development processes in this parasitic nematode.^452^ In addition to receptors and ligands, other components, such as cofactors, can also be potential targets for the manipulation of the life cycle of parasitic nematodes. For example, DAF-12 interacting protein-1 (DIP-1) was recently identified as a coactivator of DAF-12, which is indispensable for the ligand-activated transcriptional activity of DAF-12 in *Strongyloides* spp.^453^ Together, this evidence suggests that the nuclear receptor DAF-12 is a promising target for the treatment of parasitic diseases.

### Targeting nuclear receptors in reproductive diseases

Polycystic ovary syndrome (PCOS) is a type of reproductive disease that affects 5–18% of women worldwide who are of reproductive age.^454^ Interestingly, patients with PCOS are frequently associated with metabolic syndrome, such as obesity, dyslipidemia, impaired glucose tolerance or increased fasting insulin,^455^ which is closely linked to an abnormal gut microbiome. A recent study revealed that PCOS can be induced by alterations in the gut microbiome via the activation of the nuclear receptor FXR.^456^ Mechanistically, agmatine produced by B. vulgatus can activate FXR, which in turn prevents L cells from secreting glucagon-like peptide-1 (GLP-1), suggesting that FXR is a possible therapeutic target for the treatment of PCOS.^456^ Moreover, PPAR is a large nuclear receptor superfamily related to energy metabolism and is thus involved in the progression of PCOS. In fact, all three PPAR family members, including PPARα, PPARβ/δ and PPARγ, play critical roles in PCOS via multiple mechanisms, such as the regulation of lipogenesis; glucose homeostasis; and fatty acid oxidation, synthesis and storage.^457^ Given the important function of PPARs in PCOS pathogenesis, modulating the activity of PPARs profoundly affects the development of PCOS. For example, the activation of PPARγ by a polyphenolic compound, sinapic acid, was shown to ameliorate metabolic dysfunction and attenuate PCOS phenotypes in rats.^458^ Another study focusing on the downstream mechanism underlying PPAR-mediated alleviation of PCOS indicated that PPARα can bind to the promoter of FADS2, a ferroptosis-related gene, thus increasing FADS2 transcription. This event inhibits ferroptosis of granulosa cells in a dehydroepiandrosterone (DHEA)-induced PCOS mouse model, suggesting that PPARα alleviates PCOS by protecting granulosa cells from ferroptosis.^459^

Uterine leiomyomas, another name for uterine fibroids, are benign tumors of the fibrotic smooth muscle in the reproductive system. The ovarian steroid hormone estrogen, as well as its receptor ER, are involved in this disease.^460^ In addition, other nuclear receptors, such as the NR4A protein family, may also participate in the progression of uterine fibroids.^461^ NR4A restricts the proliferation of primary leiomyoma smooth muscle cells by modeling the extracellular matrix, whereas low expression of NR4A contributes to the pathogenesis of uterine fibroids. Moreover, leuprolide acetate, a GnRH analog used to treat uterine fibroids, can restore the expression of NR4A1 and NR4A3 in clinical samples, suggesting that these two NR4A family members might be additional targets of leuprolide acetate in addition to sex steroid receptors.

### Targeting nuclear receptors in renal diseases

While NRs are intimately involved in proper kidney function, they are also implicated in a variety of renal diseases, such as diabetes, acute kidney injury, and other conditions, such as aging. In the last 10 years, our understanding of renal disease etiology and progression has been greatly shaped by knowledge regarding how NRs are dysregulated in these conditions. Importantly, NRs have also become attractive therapeutic targets for the attenuation of renal diseases, and their modulation for this purpose has been the subject of intense investigation. Here, we review the roles of six key renal NRs, including PPAR, ERR, FXR, ER, LXR, and VDR, in health and disease, with an emphasis on recent findings over the last decade.

PPARs are involved in various renal diseases, both acute and chronic. PPARα-null mice treated with streptozotocin (STZ) exhibit accelerated diabetic nephropathy, including worsened albuminuria, glomerular sclerosis, mesangial matrix expansion, collagen deposition, and macrophage infiltration. PPARα agonists have been studied as potential treatments for diabetic nephropathy (DN) in rodents, with evidence supporting their renoprotective effects. Recent research by Chung et al.demonstrated that impairment of PPARα and fatty acid oxidation exacerbates kidney fibrosis during aging, with reduced PPARα expression and lipid accumulation in tubular cells.^462^ PPARα-null mice also exhibit more severe renal aging and fibrosis. Jao et al.reported that ATF6 downregulates PPARα transcription, leading to decreased fatty acid oxidation and increased lipotoxicity in proximal tubule cells, whereas ATF6-deficient mice exhibit increased PPARα expression and decreased lipid accumulation.^463^ In addition to PPARα, PPARγ has also been studied in DN, with evidence suggesting that its activity is suppressed in this disease. Unlike other PPAR family members, PPARβ/δ is ubiquitously expressed in both cortical and medullary cells. Recent studies have highlighted a potential role for PPARβ/δ in renal homeostasis. For example, Kirkby et al. demonstrated that activation of renal PPARβ/δ by endogenous prostacyclin induces vasodilation and improves blood flow, a process regulated by cyclooxygenase-2 (COX-2).^464^ This COX-2/prostacyclin/PPARβ/δ axis offers new insights into kidney vascular physiology and therapeutic possibilities. Additionally, activation of PPARβ/δ has been shown to improve renal dysfunction in a lupus mouse model by lowering blood pressure, reducing renal hypertrophy, decreasing albuminuria, and preventing renal injury.^465^

Additionally, other nuclear receptors play key roles in kidney health. ERRs are crucial regulators in the kidneys, where they help maintain metabolic balance, regulate monovalent cation homeostasis, and control blood pressure.^466^ The importance of ERRs is further highlighted by the fact that the deletion of ERRγ leads to significant mitochondrial dysfunction and progressive renal failure.^467^ For example, ERRα has been shown to protect against AKI in tubular epithelial cells, particularly in cases of cisplatin-induced AKI in mice.^468^ Compared with those in wild-type mice, in ERRα-null mice subjected to cisplatin treatment, tubular injury, a reduced mitochondrial content, abnormal mitochondrial morphology, and increased oxidative stress were observed. A recent study demonstrated that Nr4a1 was upregulated in the kidneys of mice with unilateral ureteral obstruction (UUO), and its expression was positively correlated with the degree of interstitial kidney injury and the level of fibrotic proteins. Nr4a1 may contribute to renal fibrosis by activating the p38 MAPK pathway, as treatment with the p38 MAPK inhibitor SB203580 successfully inhibited fibrotic protein expression and p38 MAPK phosphorylation.^184^ Furthermore, VDR has been shown to exert a renoprotective effect by inhibiting fibrosis. VDR translocates into the nucleus to form the VDR:RXR heterodimer, which inhibits fibrogenic gene expression in HK-2 cells exposed to high glucose and reduces fibrogenic gene expression in proximal renal tubules.^469^ In the aging kidney, the expression of FXR and TGR5 is decreased. However, a dual agonist of FXR and TGR5, INT-767, has been found to reverse age-related kidney disease in mice, demonstrating the therapeutic potential of targeting these receptors in renal aging.^470^ In conclusion, nuclear receptors play vital roles in regulating kidney function and protecting against kidney injury and fibrosis. Understanding their mechanisms and interactions could open new avenues for therapeutic strategies to treat kidney diseases, particularly those related to aging and fibrosis.

### Application of emerging technologies in NR research

Several challenges remain in targeting NRs therapeutically due to the complexity of NR signaling, tissue specificity, and the need for precise drug design. However, recent advancements in genomics, proteomics, imaging, and computational modeling have enabled more precise exploration of NR function, regulation, and their involvement in various physiological and pathological processes.

Technologies such as ChIP-chip, ChIP-Seq, and GRO-Seq have enabled unbiased, genome-wide profiling of NR binding sites, revealing new insights into NR mechanisms and their regulatory networks in breast cancer. These genomic methods have highlighted the dynamic interactions between NRs and the chromatin landscape. It is increasingly clear that chromatin, in its varying states of condensation, both regulates and is regulated by NR activity, with NR-chromatin interactions playing a major role in cell type-specific transcriptional regulation.^471^ Single-cell RNA sequencing (scRNA-seq) has further advanced our understanding of NR function by enabling gene expression analysis at the single-cell level. This technology is invaluable for investigating tissue-specific NR roles and identifying distinct signaling pathways in various cell populations, which is crucial for understanding their contributions to tissue homeostasis, development, and diseases like cancer and metabolic disorders.^472^ For instance, single-cell transcriptomics revealed the involvement of a previously uncharacterized retinoic acid receptor family member, SmRAR, in reproductive development in *Schistosoma mansoni*.^473^ CRISPR-Cas9 technology has revolutionized NR research by allowing precise manipulation of genes in vitro and in vivo. This has facilitated studies of NR roles in diseases such as type 2 diabetes, neurodegeneration, and cancer, and provide reversible and tunable control of NR expression, enabling dynamic investigation of NR function.^474,475^

Recent advances in proteomics, particularly mass spectrometry (MS), have enabled the identification and quantification of NR interactomes, providing insights into the molecular mechanisms regulating NR activity. MS also helps identify post-translational modifications like phosphorylation, acetylation, and SUMOylation, which are crucial for NR activation and signaling.^306^ Cryo-EM has emerged as a powerful tool for visualizing the 3D structure of proteins, including NRs, at near-atomic resolution. This technique has provided key insights into the conformational changes NRs undergo upon ligand binding and co-factor recruitment, essential for drug discovery, as many NRs are targeted by small molecules in diseases like cancer, diabetes, and inflammation.^476^ For example, cryo-EM has elucidated the assembly of the AR:coactivator complex and its role in coactivator assembly and transcriptional regulation.^70^ Additionally, the cryo-EM structure of the NCOA4-FTH1 interface revealed key amino acids crucial for interaction, paving the way for developing ferritinophagy modulators.^477^ These technologies, including cryo-EM and omics, have advanced our understanding of NR activation, NR-chromatin interactions, transcriptional regulation, and non-genomic effects, offering new avenues for therapeutic development.

Artificial intelligence (AI) and machine learning (ML) are increasingly applied in NR research to predict receptor-ligand interactions, identify novel signaling pathways, and design drugs targeting specific NR subtypes. These technologies analyze large datasets to uncover hidden patterns within complex biological systems, enhancing our understanding of NRs in health and disease. For example, chemical language models combined with beam search algorithms have been used for automated molecule design, leading to the development of novel inverse agonists for retinoic acid receptor-related orphan receptors (RORs) with low micromolar to nanomolar potency.^478^ AI and deep docking methodologies have also facilitated the development of VPC-260724, a novel ER-AF2 binder that inhibits ER activity and exhibits antiproliferative effects in ER-positive breast cancer models.^479^ Additionally, AI has been applied to create an adverse outcome pathway (AOP) network for chemical-induced cholestasis, facilitating automated data collection and the identification of key molecular events.^480^ Moreover, Bhimsaria and colleagues identified previously hidden binding sites of human NRs via high-throughput SELEX and a novel MinSeq Find algorithm, revealing new biological roles of NRs and their involvement in disease-associated genetic variations while providing a valuable resource for drug repurposing and precision medicine.^481^ With AI and ML, high-throughput screening (HTS) has become more efficient, enabling better prediction of drug candidates and optimization of lead compounds for clinical applications.

In vivo imaging techniques like positron emission tomography (PET) and magnetic resonance imaging (MRI) are increasingly used to study nuclear receptor (NR) activity in living organisms. These methods allow real-time tracking of NR signaling and the effects of ligands or drugs on receptor function in tissues. Live-cell imaging with fluorescent biosensors also reveals dynamic interactions between NRs and their co-regulatory proteins at the cellular level.^482^ This approach offers a quantitative, in vivo equivalent to classic radioligand binding assays.^483^ In vivo imaging helps identify when and where NRs are active during development, aging, and disease, providing insights into how ligands or receptor modulators influence these processes.^484^ Other technologies, such as organoids, 3D cell cultures, nanotechnology, and systems biology, have also advanced NR research. Notably, PROTACs (proteolysis-targeting chimeras), which degrade target proteins via the 26S proteasome, offer new possibilities for targeting challenging NRs, including orphan receptors or those without enzymatic activity. In cases of drug resistance due to NR mutations (e.g., in hormone-dependent cancers), PROTACs can bypass resistance by degrading the mutated receptors instead of inhibiting their activity.

Emerging technologies are significantly enhancing the field of nuclear receptor research by providing deeper insights into their structure, function, and regulation. The combination of high-resolution imaging, gene editing tools, and advanced computational techniques has opened new avenues for understanding how NRs control physiological processes and contribute to disease. As these technologies continue to evolve, they hold promise for the development of more precise and effective therapeutic strategies targeting nuclear receptors in a variety of disorders.

## Concluding remarks

Nuclear receptors are a crucial class of transcription factors that regulate gene expression in response to various ligands, such as hormones, vitamins and metabolic products. They play pivotal roles in processes such as metabolism, immune responses and development, making them key targets for therapeutic intervention in cardiovascular disease, cancer, metabolic disorders and inflammatory conditions. In recent years, targeted therapies aimed at NRs, such as ERs, ARs and VDRs, have shown significant promise in clinical applications, particularly in oncology and metabolic regulation. Newer nuclear receptors, such as LXR and PXR, are being investigated for their therapeutic potential, revealing novel pathways for drug development.

Despite recent advances, several challenges remain in targeting NRs for therapeutic purposes. One key difficulty is the extensive crosstalk between NRs and their signaling pathways, which can lead to synergistic or antagonistic effects, limiting the effectiveness of targeting a single receptor and causing off-target effects. Additionally, NRs exhibit tissue-specific expression and functional outcomes, making designing drugs that selectively modulate receptor activity in specific tissues or cell types challenging. The specificity of ligand-receptor interactions is another hurdle, as NRs can be activated by various small molecules, and ligand promiscuity or cross-reactivity can lead to unwanted side effects. Moreover, long-term NR modulation may result in side effects or even toxicity, such as liver damage or increased cardiovascular risk. Finally, many NRs are regulated by complex feedback mechanisms, complicating predictions about how receptor activity changes in response to treatment. These dynamic regulatory loops require careful consideration to avoid unintended consequences and therapeutic resistance.

Future research will likely focus on understanding the complex interactions of nuclear receptors in various physiological contexts, developing more selective modulators, and exploring novel strategies for more precise and selective targeting of NRs to minimize off-target effects and enhance therapeutic efficacy. Integration of data from transcriptomics, proteomics, and metabolomics will help model NR signaling networks and predict the effects of targeting a specific NR in the context of broader cellular networks. Structure-based drug design employing leverage X-ray crystallography, cryo-EM, and computational modeling will facilitate the identification or design of small molecules that specifically interact with a target NR’s ligand-binding domain, minimizing cross-reactivity. Cutting-edge technologies such as gene editing CRISPR/Cas9 technology and advanced drug delivery systems achieve tissue- or cell-specific targeting of NRs to minimize off-target effects and enhance therapeutic efficacy. Noninvasive techniques to monitor the activity of NRs via specifically binding imaging agents to activated nuclear receptors or linking reporter genes (e.g., luciferase, GFP) to NR-responsive promoters will allow real-time visualization and quantification of receptor activity in preclinical models. In addition, the fact that NRs are key host sensors of microbial signals offers a promising avenue for gut microbiota-targeted drug discovery by leveraging NR-mediated environmental adaptations.^485–487^ For example, FXR plays a pivotal role in modulating the gut microbiota and is thereby involved in various biological processes, including the metformin response,^488^ bile acid homeostasis,^489^ and core metabolic functions.^490^ Overall, with the advancement of these technologies, new breakthroughs will be made in the discovery of novel drugs that target NRs.

## Data Availability

Not applicable.
